# The tumour immune microenvironment is enriched but suppressed in vestibular schwannoma compared to meningioma: therapeutic implications for *NF2*-related schwannomatosis

**DOI:** 10.1186/s40478-025-02176-9

**Published:** 2025-12-23

**Authors:** Grace E. Gregory, Michael J. Haley, Adam Paul Jones, Leo A. H. Zeef, D. Gareth Evans, Andrew T. King, Pawel Paszek, Kevin N. Couper, David Brough, Omar N. Pathmanaban

**Affiliations:** 1 Division of Neuroscience, School of Biological Sciences, Faculty of Biology, Medicine and Health, University of Manchester, Manchester Academic Health Science Centre, Manchester, UK; 2 Geoffrey Jefferson Brain Research Centre, The Manchester Academic Health Science Centre, Northern Care Alliance NHS Foundation Trust, University of Manchester, Manchester, UK; 3 The Lydia Becker Institute of Immunology and Inflammation, University of Manchester, Manchester, UK; 4 Bioimaging Core Facility, Faculty of Biology, Medicine and Health, University of Manchester, Manchester, M13 9PT UK; 5 Edwin L. Steele Laboratories, Department of Radiation Oncology, MassaCtts General Hospital and Harvard Medical School, Boston, MA 02114 USA; 6 Bioinformatics Core Facility, University of Manchester, Manchester, UK; 7 Division of Evolution, Infection and Genomic Sciences, School of Biological Sciences, Faculty of Biology, Medicine and Health, University of Manchester, Manchester Academic Health Science Centre, Manchester, UK; 8 Division of Cardiovascular Sciences, School of Biological Sciences, Faculty of Biology, Medicine and Health, University of Manchester, Manchester Academic Health Science Centre, Manchester, UK; 9 Department of Neurosurgery, Manchester Centre for Clinical Neurosciences, Salford Royal Hospital, Northern Care Alliance NHS Foundation Trust, Stott Lane, Salford, M6 8HD UK; 10 Institute of Fundamental Technological Research, Polish Academy of Sciences, Pawinskiego 5B, 02-106 Warsaw, Poland; 11 Division of Immunology, Immunity to Infection and Respiratory Medicine, Faculty of Biology, Medicine and Health, University of Manchester, Manchester, UK

**Keywords:** NF2, *NF2*-related schwannomatosis, Tumour microenvironment, Inflammation, Vestibular schwannoma, Meningioma, Skull base neoplasm, Tumour associated macrophages, CD8 T cells, TAM

## Abstract

Currently there are no therapeutic agents that are effective against both vestibular schwannoma and meningioma, the two most common tumour types affecting patients with the rare tumour predisposition syndrome *NF2*-related schwannomatosis. This study aimed to characterise the similarities and differences in the tumour immune microenvironments of meningioma and vestibular schwannoma to identify potential therapeutic targets viable for both tumour types. Publicly available bulk Affymetrix expression data for both meningioma (*n* = 22) and vestibular schwannoma (*n* = 31) were used to compare gene expression and signalling pathways, and deconvolved to predict the abundance of the immune cell types present. Publicly available single cell RNA sequencing data for both meningioma (*n* = 6) and vestibular schwannoma (*n* = 15) was used to further investigate specific T cell and macrophage subtypes for their signalling pathways, gene expression, and drug targets for predicted drug repurposing in both tumour types. Immune cells comprised a larger proportion of the vestibular schwannoma tumour microenvironment compared to meningioma and included a significantly higher abundance of alternatively activated macrophages. However, these alternatively activated macrophages, alongside other immune cell subtypes such as CD8 + T cells and classically activated macrophages, were predicted to be more active in meningioma than vestibular schwannoma. Despite these differences, T cells and tumour associated macrophages of both vestibular schwannoma and meningioma shared drug-target kinases amenable to drug repurposing with Food and Drug Administration (FDA) drugs approved for other conditions. These include bosutinib, sorafenib, mitoxantrone, and nintedanib which are yet to be clinically investigated for vestibular schwannoma or meningioma. Drug repurposing may offer an expedited route to the clinical translation of approved drugs effective for treating both meningioma and vestibular schwannoma to benefit *NF2*-related schwannomatosis patients.

**Supplementary Information:**

The online version contains supplementary material available at 10.1186/s40478-025-02176-9.

## Introduction

The rare tumour predisposition syndrome *NF2*-related schwannomatosis (*NF2*-SWN) is characterised by the development of multiple central and peripheral nervous system tumours. Over 95% of those with *NF2*-SWN develop bilateral vestibular schwannoma (VS), which are neoplasms formed of over-proliferating Schwann cells in the eighth cranial nerve [35]. Additionally, *NF2*-SWN patients have a lifetime incidence of around 80% for meningioma, developing from tumourigenic arachnoid cap or dural border cells of the meningeal membranes [14, 35, 42].

With heterogeneity in the natural history of *NF2*-SWN, its symptomatic presentation is also highly varied and related to genetic severity [18]. Patients commonly experience sensorineural hearing loss, tinnitus, vertigo, and balance dysfunction due to the presence of VS tumours, alongside less frequent symptoms from additional tumour burden such as seizures, weakness and wasting, pain, and loss of vision [11, 35]. Despite this, management options for people with *NF2*-SWN are currently limited to monitoring by MRI, surgical excision, radiotherapy, and for some cases, the off-label use of the anti-angiogenesis drug, bevacizumab [28, 36, 39], and more latterly a potential role for brigatinib in *NF2*-related meningioma [37]. However, there are no approved drug options available to these patients and, for reasons yet to be fully understood, bevacizumab is ineffective for meningioma treatment [27, 33]. Therefore, there is an urgent need to identify new therapeutic targets for the treatment of *NF2*-SWN patients, especially using agents that are effective across the different tumour-types.

The majority of studies co-investigating VS and meningioma have focussed on therapeutic testing in vitro, in vivo, and in clinical trials without first investigating similarities and differences between these tumour types [6, 21, 31, 43]. The few studies that have directly assessed VS and meningioma indicate similarities in *NF2* gene pathogenic variants and in gene expression related to angiogenesis and tumour suppression (*PDGFD*, *CDH1* and *SLIT2*) [30, 48]. Despite the increasing evidence of the role of immune cells in both meningioma and VS growth, [14, 19, 38, 50] as of yet no studies have compared the tumour immune microenvironments of meningioma and VS.

This study directly evaluated the similarities and differences between VS and meningioma, with a focus on the immune compartment of the tumour microenvironment. Utilising publicly available bulk and single cell transcriptomic datasets, this study provides a foundation for the pre-clinical testing of 10 FDA and National Institute for Health and Care Excellence (NICE)-approved therapeutics for the treatment of both meningioma and VS, with the potential for fast-tracking to the clinic as valuable *NF2*-SWN treatments.

## Materials and methods

### Affymetrix microarray data

For controls, VS, and meningioma samples, publicly available Affymetrix microarray data from GSE54934 were extracted from Gene Expression Omnibus (GEO). Clinical information on patient samples are found in Supplementary Table 1, the GSE54934 GEO repository and associated publication [49]. Microarray data pre-processing required performing Robust Multichip/multi-array Analysis (RMA) using the R Bioconductor package, ‘affycoretools’ with subsequent annotation using the U219 array database ‘pd.hugene.1.0.st.v1’ [7, 29]. Differential gene expression analysis was conducted in R using the ‘limma’ package with empirical Bayes to analyse six groups: control vestibular and eighth cranial nerve *n* = 2, VS tissue (sporadic *n* = 28 and *NF2*-SWN *n* = 3), control meningeal tissue *n* = 3, and meningioma tissue (sporadic *n* = 20 of which *n* = 18 World Health Organisation (WHO) grade 1 and *n* = 2 WHO grade 2, and *NF2*-SWN WHO grade 1 meningioma *n* = 2) [40]. The Benjamini–Hochberg method was used to control *p* for false discovery rate (FDR) [5]. Differentially expressed genes (DEG) were considered when the FDR-adjusted *p* ≤ 0.01 with a fold change of ≥ 2 or ≤ -2. Principal component analysis (PCA) was performed on all six groups. Where multiple probes were present for each gene the expression was averaged to reduce probe set variation per case. Hierarchical clustering of the six groups by relative mean gene expression used one minus Pearson’s correlation.

### Affymetrix gene expression deconvolution

To deconvolve the bulk Affymetrix gene expression data into predicted immune cell proportions, CIBERSORTx was utilised on meningioma and VS (including both sporadic and *NF2*-SWN cases) [34, 44]. The validated LM22 signature matrix for the tumour immune microenvironment was applied to the annotated Affymetrix RMA gene expression data for GSE54934 in transcripts per million [8]. Batch correction was enabled in ‘bulk mode’ in absolute at 1000 permutations, using the associated LM22 Source Gene Expression Profile (GEP) file. Relative abundance for each cell type was compared between meningioma and VS cases. The normality of distribution of the data were determined by Shapiro–Wilk test, followed by Mann–Whitney U test with Benjamini–Hochberg adjustment to control for FDR. Statistical significance was considered when the FDR-corrected *p* < 0.05.

### Single cell RNA sequencing data

Publicly available single-cell RNA sequencing (RNA-seq) data from six sporadic meningioma (*n* = 2 WHO grade 1, *n* = 3 grade 2, and *n* = 1 grade 3) and 15 sporadic VS were obtained from GEO repositories GSE183655 and GSE216783 respectively, where patient clinical information is described in Supplementary Table 1 and can be found in the GEO repositories and associated publications [4, 10]. Data were pre-processed using python package Scanpy to compute per-cell and per-gene quality control to remove cells of poor quality and doublets, leaving mitochondrial genes in the data to help identify changes in mitochondrial function (however, poor quality cells with > 20% mitochondrial gene expression were removed) [51]. Data were normalised with log plus one transformation, with PCA for dimensionality reduction on the top 1500 highly variable genes, and Python package Harmonypy was utilised for dataset integration batch correction by individual sample [23].

Unsupervised Leiden clustering defined 19 cell clusters in the normalised dataset. These were condensed or further subclustered into biological cell types annotated according to semi-supervised naming by DecoupleR package, using Over Representation Analysis and Rank Genes Groups (RGG) to indicate cell type by the top 5% of genes expressed [3]. Final annotated cell type clusters were visualised by Uniform Manifold Approximation and Projection (UMAP). RGG was employed using the Wilcoxon method with Benjamini–Hochberg FDR adjustment for single cell-based DEG analysis of genes between meningioma and VS cell types of interest, namely macrophages and T cells. DEGs were determined when *p* < 0.05 with fold change of ≤ -2 or ≥ 2. Relative abundance of cell types was compared between meningioma and VS using Shapiro Wilk normality test followed by Mann–Whitney U test with Benjamini–Hochberg adjustment, where significance was determined at *p* < 0.05. Pseudo-bulk data of meningioma T cells, meningioma macrophages, VS T cells or VS macrophages was generated per single cell RNA-seq sample, with PCA computed, and pseudo-bulk DEG analysis completed using DESeq2 package applying the Wald test with Benjamini–Hochberg FDR adjustment with significance when *p* < 0.05 with fold change of ≤ -2 or ≥ 2.

### Ingenuity pathway analysis

FDR-adjusted *p* for DEG lists with their absolute fold-changes were loaded into Ingenuity Pathway Analysis (IPA) (Qiagen) for Affymetrix and pseudo-bulk data. The defaults for the ‘Core analysis’ function were applied to retrieve significantly dysregulated canonical pathways and pathway categories enriched in VS or meningioma against their control tissues from Affymetrix, as well as the direct comparison between pseudo-bulk VS and meningioma for T cells and macrophages according to the Ingenuity Pathway Knowledge Base (IPKB). The ‘Analysis Match’ function was used for drug repurposing hypothesis generation by filtering for the Library of Integrated Cellular Signatures (LINCS) program for drug/control expression data. LINCS data was sorted by lowest z-score compared to sporadic VS/control and sporadic meningioma/control to identify FDA/NICE approved drugs from the top 200 LINCS studies that were predicted to induce gene expression changes that could revert VS and meningioma toward the gene expression of their control tissues. Direct chemical-protein interactions between significantly different (Wald test with Benjamini–Hochberg FDR adjustment *p* < 0.05 with fold change ≤ − 2 or ≥ 2) and non-significantly different IPA-annotated kinases and the list of FDA/NICE approved drugs according to the IPKB were established.

## Results

### Vestibular schwannoma exhibit stronger immune-based gene signatures than meningioma

**Fig. 1:**
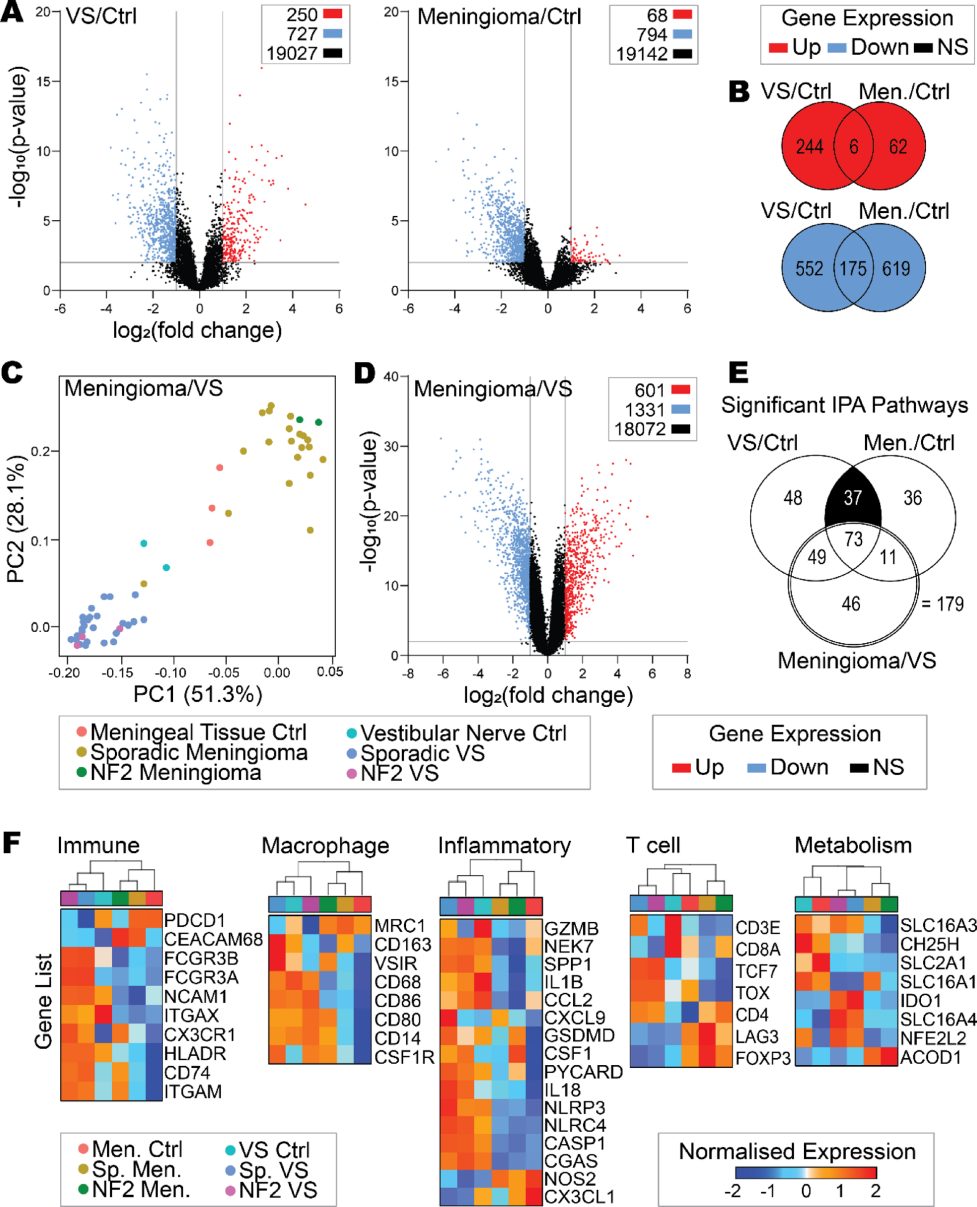
Vestibular schwannoma have more immune rich gene expression profiles than meningioma. Data acquired from GSE54934 from Gene Expression Omnibus. **A** Volcano plots of differentially expressed genes (DEG) between VS (sporadic *n* = 28 and NF2 *n* = 3) and control vestibular nerve (*n* = 2) or meningioma (sporadic *n* = 20 and NF2 *n* = 2) and control meningeal tissue (*n* = 3). DEGs defined as *p* ≤ 0.01 with fold change of ≤ -2 or ≥ 2. **B** Co-overexpressed or co-underexpressed genes in VS and meningioma versus their respective control tissues. **C** Principal component analyses (PCA) for gene expression dimensionality reduction displaying all VS, meningioma and control samples. **D** Volcano plot of DEGs from direct meningioma and VS comparison. **E** The number of significant signalling pathways identified in VS or meningioma compared to nerve, or VS and meningioma direct comparison using Ingenuity Pathway Analysis (IPA). *p* ≤ 0.05. **F** Gene expression associated with immune profile, macrophage, inflammatory profile, T cell, and metabolism determined using IPA and literature search. One-minus Pearson correlation for hierarchical clustering. Genes named by Human Gene Nomenclature. Abbreviations: meningioma (Men.), vestibular schwannoma (VS), sporadic (Sp.), control (Ctrl), principal component (PC), not significant (NS)

Initially the GSE54934 dataset was used to investigate similarities and differences in bulk gene expression between VS and meningioma, which contained *n* = 28 sporadic VS, *n* = 3 *NF2*-SWN VS and *n* = 2 control vestibular nerve as well as *n* = 20 sporadic meningioma, *n* = 2 *NF2*-SWN meningioma and *n* = 3 control meningeal tissue (clinical characteristics by sample are found in Supplementary Table 1). DEGs were identified between the tumour samples and their control tissues (vestibular nerve and meningeal tissue, respectively) (Fig. 1A). DEG analysis identified 250 and 68 significantly overexpressed genes in the VS and meningioma tumour samples respectively compared to their control tissues, including six significantly co-overexpressed genes: *COL1A*, *ERBB2*, *SLFN12*, *SLIT2*, *PDGFD* and *CDH1* (Fig. 1A, B, and Supplementary Table 2). Additionally, when compared against their control tissues, VS and meningioma had 727 and 794 significantly under-expressed genes respectively, including 175 overlapping significantly co-underexpressed genes (Supplementary Table 2). These significantly co-over and co-underexpressed genes indicated similarities in meningioma and VS verses their control tissues as these genes were enriched in pathways involved in cellular stress, neuronal/nervous system signalling, growth factor signalling, and growth, proliferation, and development (Supplementary Table 3).

Next, a direct comparison of the bulk gene expression signatures between meningioma and VS tumours was completed. Overall, meningioma and VS samples clustered separately by principal component 1 on PCA indicating identifiable gene expression differences between the two tumour types (Fig. 1C), with a total of 1932 (5%) significant DEGs between meningioma and VS relating to 179 significantly different IPA pathways (Fig. 1D, E). The top three IPA pathways significantly dysregulated between meningioma compared to VS were associated with axonal guidance, myelination, and fibrosis (Supplementary Fig. 1).

However, similarities remained between meningioma and VS including the 18,072 genes (95%) that were not significantly differentially expressed between the two tumour types (Fig. 1D). There were 37 IPA pathways significantly dysregulated in the tumours when compared against their control tissues, but importantly these were not significantly different when directly comparing meningioma and VS (Fig. 1E and Supplementary Fig. 2). These represented pathways that may be amenable to drug targeting effective for both meningioma and VS and include apelin and G protein-coupled receptor signalling, calcium and neurotransmitter signalling, nitric oxide signalling, adherens junction remodelling, and vesicle transport (Supplementary Fig. 2).

To investigate specific immune microenvironment-related genes, the bulk expression of genes of interest between meningioma, VS and their control tissues were assessed in Fig. 1F. *NF2*-SWN and sporadic cases of each tumour type showed overall similar profiles in the expression of the immune microenvironment genes. Differences in the expression of genes relating to innate immunity (such as *NLRP3*, *IL-18* and *IL-1B*) between meningioma and VS were identified, with VS having a more immune-rich, inflammatory profile than meningioma overall (Fig. 1F). Despite this, there was a noted increase in *FOXP3* expression in meningioma compared to the control tissues and VS tumours, and genes associated with T cell exhaustion in VS (*TOX* and *TCF7*).

Together, these data indicated similarities between *NF2*-SWN meningioma or VS with their sporadic counterparts, as well as similarities in cellular growth and proliferation between meningioma and VS overall when compared against their control tissues. However, differences between meningioma and VS were identified in the tumour microenvironment relating to fibrosis, myelination, innate inflammation, and T cell activation states.

### VS are composed of a larger immune compartment than meningioma

Bulk Affymetrix gene expression was deconvolved to predict the relative abundance of cells within the immune compartment of VS and meningioma tumour microenvironments. Most of the predicted lymphoid populations were significantly more abundant in the immune cell compartment of meningioma compared to VS (plasma cells, mast cells, natural killer (NK) cells and T cells). In contrast, myeloid populations such as dendritic cells, neutrophils, monocytes, and macrophages were predicted to be significantly increased in VS compared to meningioma (Supplementary Figs. 3 and 4). T cells were further subclassified based on their specific expression profiles. CD4 T cells were predicted to make up significantly more of the immune compartment of VS than meningioma. However, T regulatory, CD8 and other T cells were significantly increased within the meningioma immune compartment compared to that of VS (Supplementary Fig. 4). These data indicated that meningioma and VS were enriched in different T cell subtypes. *NF2*-SWN and sporadic forms of both tumour types showed similar predicted abundance of immune cells from the bulk deconvolution (Supplementary Fig. 4).

**Fig. 2:**
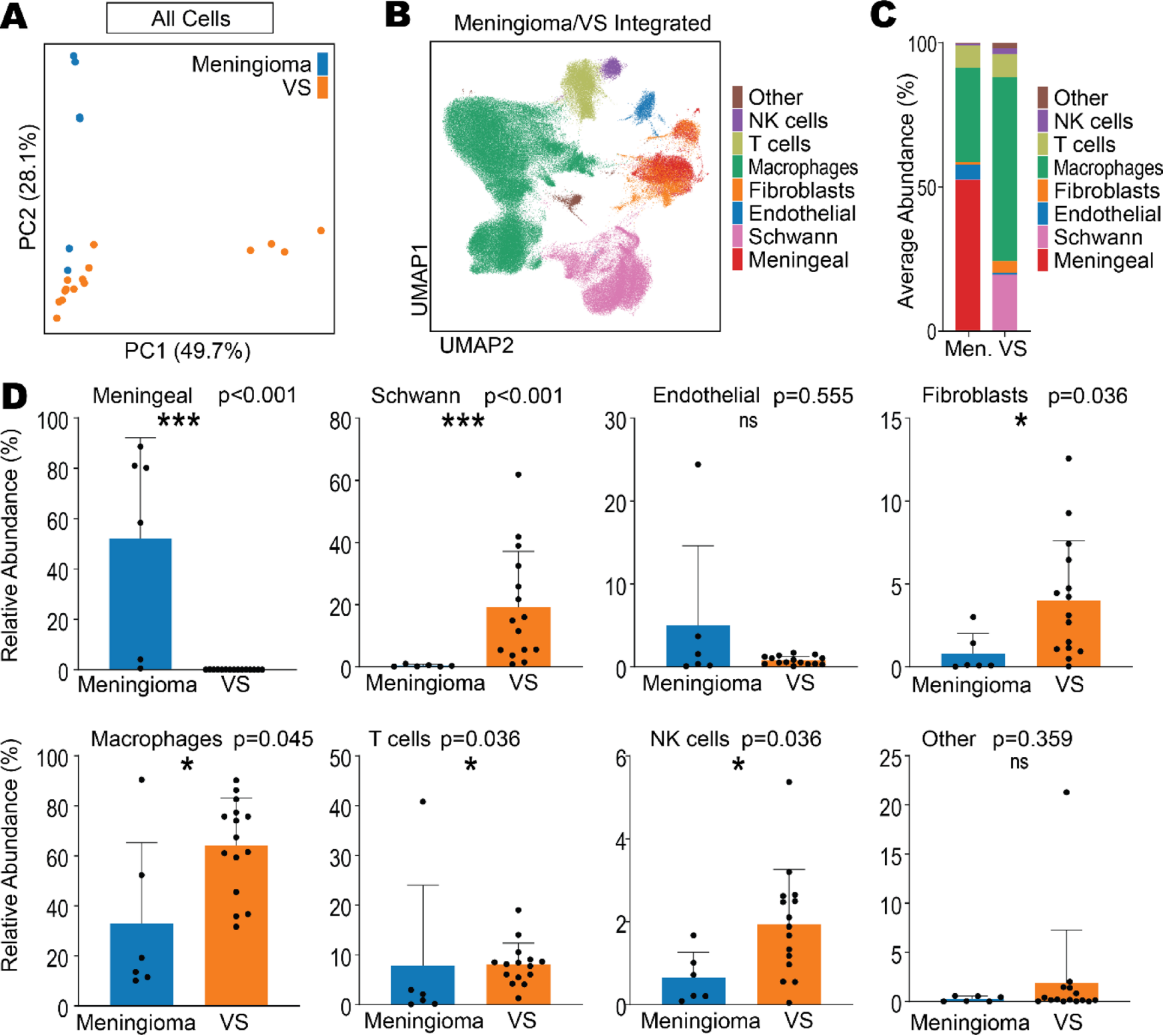
VS are composed of a larger immune compartment than meningioma. Single cell RNA sequencing data acquired from GSE183655 and GSE216783 from Gene Expression Omnibus containing meningioma (*n* = 6) and VS samples (*n* = 15), respectively. **A** Principal component analysis (PCA) of meningioma and VS samples. **B** Leiden clustering of all single cells integrated from meningioma and VS samples annotated into broad cell types, visualised by UMAP. **C** Averaged relative abundance of cell types within the tumour microenvironments of meningioma and VS. **D** Relative abundance of specific cell types in meningioma and VS cases. Shapiro Wilk normality test followed by Mann–Whitney U test with Benjamini–Hochberg adjustment where significance was determined at * *p* < 0.05, ** *p* < 0.01, *** *p* < 0.001. Abbreviations: meningioma (Men.), vestibular schwannoma (VS), principal component (PC), not significant (ns)

While bulk deconvolution was limited to the immune compartment of the tumours (as per the use of the validated, non-specific LM22 CIBERSORTx signature matrix) and could not consider the abundance of tumour/stromal components, the use of single cell RNA-seq overcomes this limitation by providing cell counts and gene expression profiles at the single cell level. Therefore, GSE216783 VS and GSE183655 meningioma datasets were used to investigate similarities and differences in single cell gene expression between VS and meningioma, which contained *n* = 15 sporadic VS (including *n* = 12 VS with known pathogenic variants in *NF2*), and *n* = 6 sporadic meningioma (including *n* = 3 meningioma with 22q loss), respectively (where full clinical characteristics by sample are found in Supplementary Table 1). After integrating and preprocessing the single cell RNA-seq GSE183655 meningioma and GSE216783 VS datasets together, PCA revealed no distinct clustering of meningioma from VS, indicating similarities in the overall expression profiles of cells in the whole tumour microenvironments (Fig. 2A). The unique cell populations within the tumour microenvironments of meningioma and VS (visualised by UMAPs in Supplementary Fig. 5A), were annotated with a combination of the semi-supervised package DecoupleR and manually by common marker genes from the literature and DecoupleR (Supplementary Fig. 5B-C) [3]. Final integrated, annotated populations were visualised by UMAP (Fig. 2B).

Average abundance of tumour/stromal components (meningeal, Schwann, endothelial, fibroblasts and other cells) and immune compartment (macrophages, T cells and NK cells) was assessed for meningioma and VS (Fig. 2C). The majority of the cells comprising meningioma were neoplastic meninge-derived cells (52%) referred to as “meningeal cells”, whereas only 19% of the cells identified in the VS tumours were neoplastic Schwann cells (Fig. 2C, D). No significant differences were noted in the abundance of endothelial cells between the two tumour types, however VS contained a significantly greater proportion of fibroblasts. Regarding the relative proportion of the immune compartments, meningioma were composed of a smaller immune compartment (41%) than VS (74%) (Fig. 2C, D). In contrast, immune cells (and specifically macrophages) primarily comprised the bulk of the cells present in VS, significantly more than in meningioma. The relative abundance of tumour microenvironment cell types by individual meningioma and VS samples in the GSE183655 and GSE216783 datasets are shown in Supplementary Fig. 6.

Vestibular schwannoma and meningioma samples were categorised by clinical information by GSE216783 and GSE183655 dataset authors Barrett (2024) [4] and Choudhury and Raleigh (2022) [10] respectively, for skull base location, 22q status, immune-enriched or other meningioma subtype and sex. By cell type abundance, meningioma subcategories were not significantly different from one another (Supplementary Fig. 7A-D: no difference between skull base and non-skull base meningioma, 22q + and 22q- meningioma, immune-enriched and other methylation group meningioma, nor male and female meningioma). Interestingly, when comparing VS against categorised meningioma, the cell type abundance of VS was more similar to immune-enriched meningioma than other methylation group meningioma (Supplementary Fig. 7C). When enumerating significantly over- and under-expressed genes between VS and categorised meningioma (Supplementary Fig. 8A-E), this similarity was reinforced as there were fewer significant DEGs between VS and immune-enriched meningioma (2737 over- and 1588 under-expressed DEGs) compared to VS versus other methylation group meningioma (4189 over- and 2195 under-expressed DEGs). After accounting for inter-tumoural variation by removing DEGs that were found between all VS and all meningioma, the top 10 significant IPA signalling pathways that were enriched in the remaining relevant DEGs (Supplementary Fig. 8D) were established (Supplementary Table 4). These included immune-relevant pathways related to cytokine storms, Th1 and Th2 activation, neutrophil degranulation, classical activation of macrophages, and alternative activation of macrophages which were upregulated in VS compared to other methylation type meningioma. However, immune-relevant pathways were not found in the top 10 IPA signalling pathways significantly different between VS and immune enriched meningioma, instead finding differences in cell cycle-relevant pathways (mitotic prometaphase, metaphase, anaphase, checkpoints) thus indicating more similarity in their immunological signalling and tumour immune microenvironment.

### VS CD4 + T cells are more active than in meningioma, whereas VS CD8 + T cells are less active

**Fig. 3:**
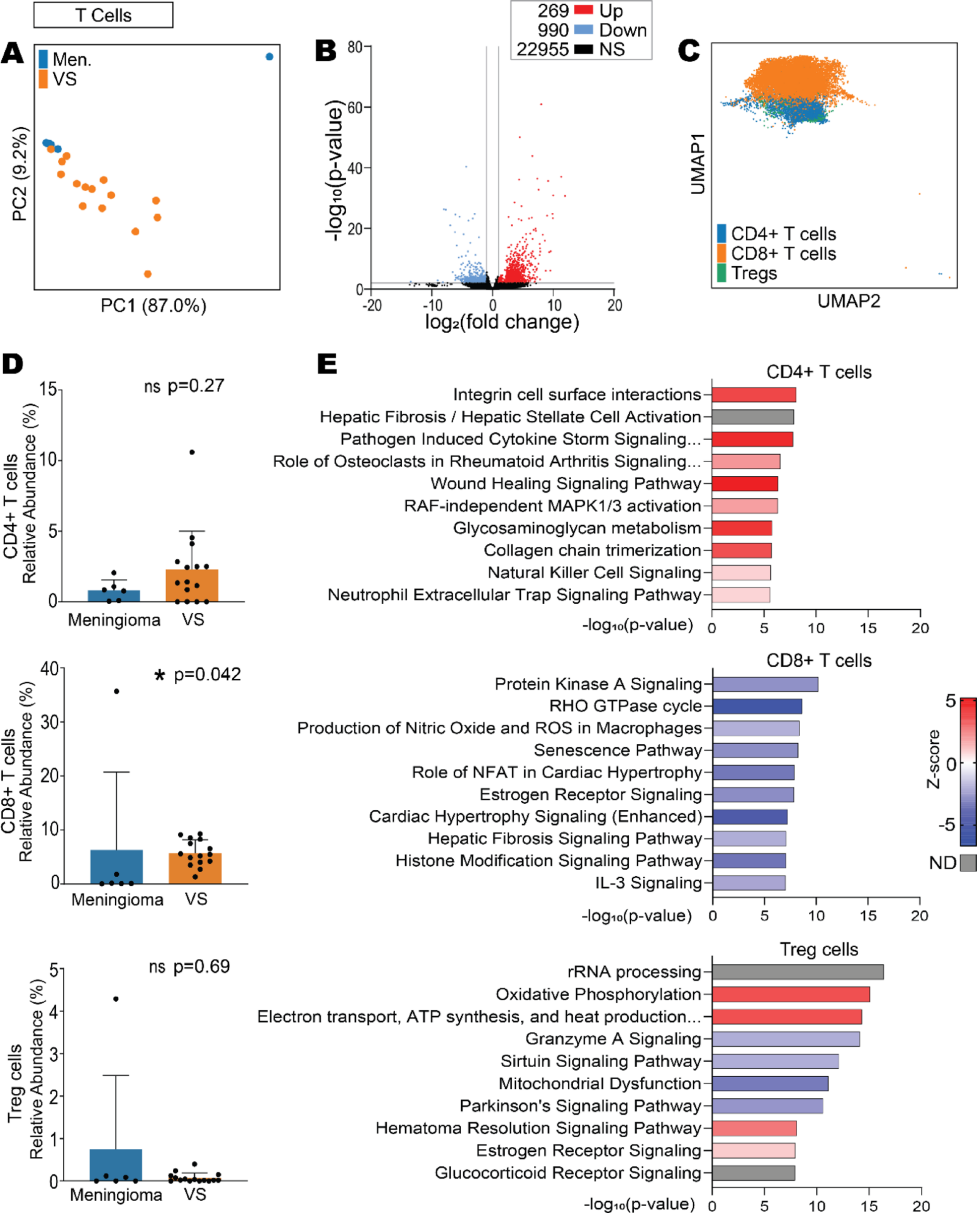
CD4 + T-cells are activated and CD8 + T-cells are suppressed in VS compared to meningioma. Single cell RNA sequencing data acquired from GSE183655 and GSE216783 from Gene Expression Omnibus containing meningioma (*n* = 6) and VS samples (*n* = 15), respectively. **A** Principal component analysis (PCA) of pseudo-bulk data of VS and meningioma samples’ T cells. **B** DEG volcano plot of pseudo-bulk VS T cells compared to meningioma T cells where DEG were defined using the Wald test with Benjamini–Hochberg adjustment, significance as *p* < 0.05 and fold change > 2 or < 2. **C** UMAP of CD4 + , CD8 + and T regulatory (Treg) cells. **D** Relative abundance of T cell subtypes: CD4 + , CD8 + and Treg cells. Shapiro Wilk normality test followed by Mann–Whitney U test with Benjamini–Hochberg adjustment where significance was determined at* p* < 0.05. **E** Top 10 significantly dysregulated IPA pathways from pseudo-bulk VS CD4 + , CD8 + and Treg cells compared to meningioma. Abbreviations: Differentially expressed genes (DEG), meningioma (Men.), vestibular schwannoma (VS), principal component (PC), not significant (NS), Ingenuity Pathway Analysis (IPA), no data available (ND)

Due to the differences noted in T cell subtypes predicted from the bulk data deconvolution, T cell subtypes were explored in the single cell RNA-seq data in the first instance. Firstly, gene expression dimensionality reduction by PCA of the total T cells revealed separation of the meningioma and VS samples (Fig. 3A) indicating differences in T cell global gene expression profiles. Furthermore, there were 1259 significant DEGs (269 overexpressed and 990 under-expressed) between the T cells of VS compared to meningioma (Fig. 3B). Specific T cell subtypes (CD4 + , CD8 + and Treg cells) in the integrated meningioma-VS dataset were visualised by UMAP (Fig. 3C). These populations were present in both meningioma and VS tumours (Supplementary Fig. 9A). This was confirmed when enumerating the relative abundance of T cell subtypes in VS and meningioma, with significant differences noted in CD8 + T cells (Fig. 3D). However, as noted previously, the variability in the meningioma samples’ immune compositions was greater than that of the VS likely due to the ‘immunogenic’ or tumour/stromal subtypes of meningioma.

Interestingly, differences in the gene expression profiles between VS and meningioma in the subtypes of T cells were identified, as well as in their significantly dysregulated signalling pathways. Notably, T regulatory cells (Treg cells) in VS displayed significantly increased expression of mitochondrial (MT-) related genes compared to meningioma. Genes involved in protein synthesis, transport, and RNA degradation were enriched in the top 10 significantly under-expressed genes in VS CD4 + , CD8 + and Treg cells compared to meningioma (Supplementary Fig. 10). Interestingly, pathway analysis based on DEGs revealed CD4 + T cells in VS compared to meningioma were predicted to be significantly more active in their signalling pathways, including those involved in cell surface interactions, activation, and cytokine release (Fig. 3E). In contrast, CD8 + T cells showed a distinct suppression in their signalling pathways in VS compared to meningioma, including those pathways involved in cell signalling, stress responses, and senescence.

### Classically and alternatively activated TAMs in VS are less active than in meningioma

**Fig. 4:**
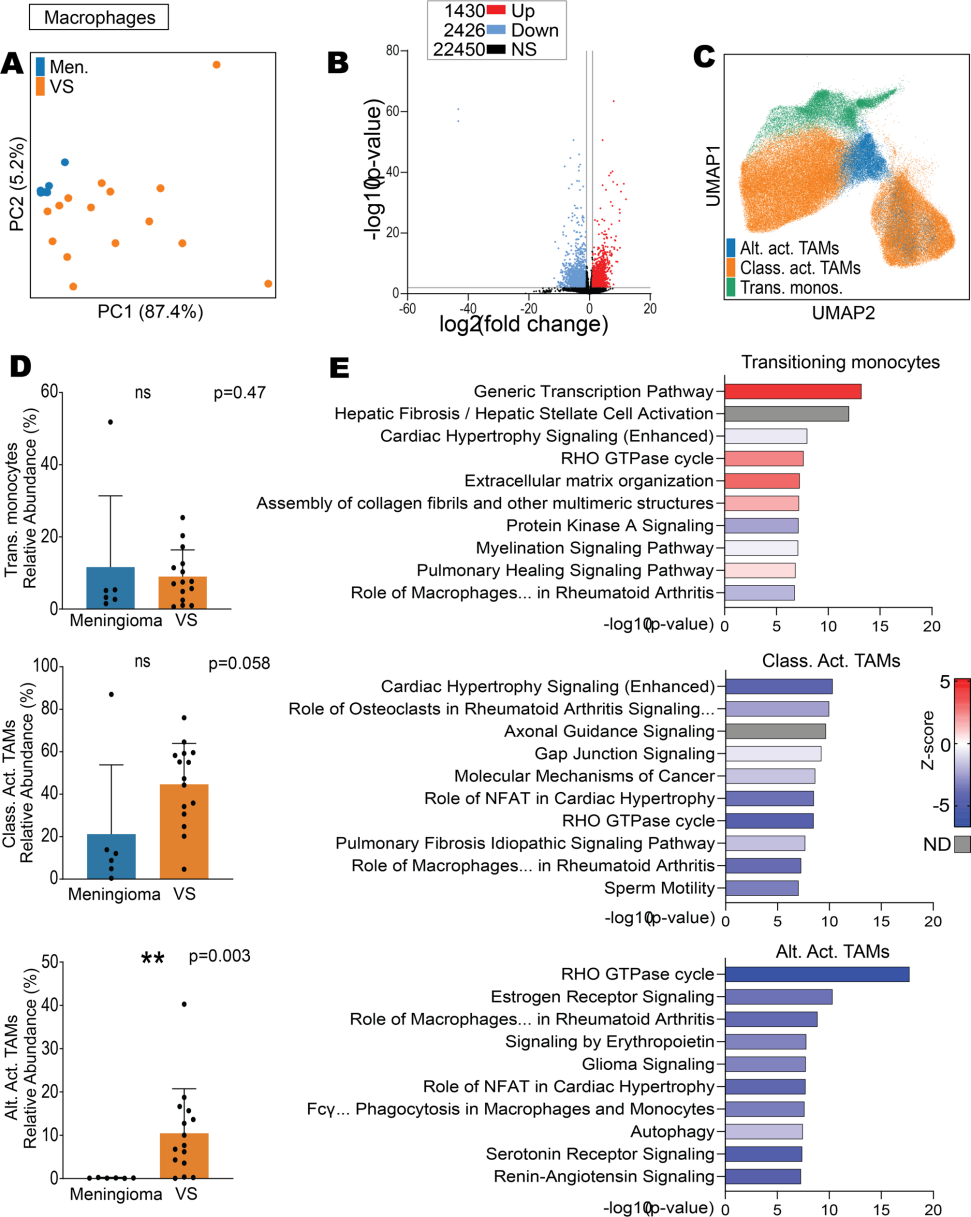
VS classically activated TAMs and alternatively activated TAMs showed predicted suppression compared to meningioma TAMs. Single cell RNA sequencing data from the macrophage cluster only, original data acquired from GSE183655 and GSE216783 from Gene Expression Omnibus containing meningioma (*n* = 6) and VS samples (*n* = 15), respectively. **A** Principal component analysis (PCA) of pseudo-bulk data of VS and meningioma samples’ macrophages. **B** DEG volcano plot of pseudo-bulk VS macrophages compared to meningioma macrophages where DEGs were defined using the Wald test with Benjamini–Hochberg adjustment, significance as *p* < 0.05 and fold change > 2 or < 2. **C** UMAP of myeloid subtypes: transitioning monocytes (trans. monos.), classically activated TAMs (class. act. TAMs) and alternatively activated TAMs (alt. act. TAMs). **D** Relative abundance of transitioning monocytes, classically activated TAMs, and alternatively activated TAMs between meningioma and VS. Shapiro Wilk normality test followed by Mann–Whitney U test with Benjamini–Hochberg adjustment where significance was determined at * *p* < 0.05, ** *p* < 0.01. **E** Top 10 significantly dysregulated IPA pathways from pseudo-bulk VS myeloid subtypes compared to meningioma. Abbreviations: Differentially expressed genes (DEG), meningioma (Men.), tumour associated macrophage (TAM), vestibular schwannoma (VS), principal component (PC), not significant (ns), Ingenuity Pathway Analysis (IPA), no data available (ND)

From the single cell RNA-seq data, the macrophages of meningioma and VS were also compared for their gene expression, subtypes, and enriched signalling pathways. Firstly, gene expression dimensionality reduction by PCA of the macrophages revealed separate clustering of the meningioma from the VS samples, thus indicating differences in gene expression between the macrophages of the two tumour types (Fig. 4A). This was confirmed with DEG analysis revealing there were 3856 significant DEGs (1430 overexpressed and 2426 under-expressed) between the macrophages of VS compared to meningioma (Fig. 4B). The myeloid subtypes (transitioning monocytes, alternatively activated tumour associated macrophages (TAM), and classically activated TAMs) in the meningioma-VS integrated dataset, which were found in both the meningioma and VS samples, were visualised by UMAP (Fig. 4C and Supplementary Fig. 9B).

When calculating the relative abundance of macrophage subtypes by activation states, there was no significant difference between VS and meningioma for transitioning monocytes and classically activated TAMs (Fig. 4D). However, the tumour microenvironment of VS contained a significantly higher relative abundance of alternatively activated TAMs compared to meningioma (*p* = 0.003).

Differences in the gene expression profiles between VS and meningioma in their macrophage subtypes were identified, but also in their significantly dysregulated signalling pathways. There was an overlap in five of the top 10 significantly under-expressed genes and two of the top 10 significantly overexpressed genes in VS transitioning monocytes and VS alternatively activated TAMs compared to meningioma (Supplementary Fig. 11). These seven similarly expressed genes (namely *RPL17*, *GABARAP*, *EEF1G*, *RNASEK*, *NME2*, *PTP4A1*, and *FKBP2)* are involved in protein synthesis, protein transport, RNA degradation, ATP homeostasis, tyrosine phosphate-driven signalling, and T cell immunosuppression. This strong overlap in significant gene expression profiles indicated that the transitioning monocytes were closer in functional activity to the alternatively activated TAMs than the classically activated TAMs. However, pathway analysis revealed that both classically and alternatively activated TAMs in VS compared to meningioma were significantly less active in their signalling pathways (Fig. 4E). As such, macrophages may have different functions in meningioma and VS, with different influences on their tumour microenvironments.

### VS and meningioma share potential drug repurposing candidates

Considering the common co-occurrence of VS and meningioma tumours in *NF2*-SWN patients, repurposing known FDA/NICE approved drugs that could be effective for both types of tumours would be highly valuable. LINCS is a gene expression database for small molecule-treated cell lines designed to characterise the gene expression changes induced by specific drug treatments, including FDA and NICE approved agents. By matching the gene expression profiles of LINCS’s drug-treated cells to the gene expression difference between a tumour and its control tissue (by filtering on lowest IPA z-score), potential therapeutics to revert tumours to a control tissue state can be identified.

From the top 200 drug treatment LINCS studies with the lowest IPA z-scores when compared against meningioma/control and VS/control Affymetrix data, only 10 compounds were approved by FDA and NICE (Table 1). Notably, nine of the 10 drugs primarily target kinases and receptor tyrosine kinases (RTK) (with the exception of mitoxantrone that targets topoisomerase) thus highlighting kinases as potential therapeutic targets within VS and meningioma amenable to drug repurposing. A number of the 10 drugs have been or are currently under clinical investigation for VS and meningioma in *NF2*-SWN patients (crizotinib, lapatinib, and everolimus), sporadic VS (crizotinib, lapatinib, and everolimus) and sporadic meningioma (everolimus, trametinib, sunitinib, and gefitinib) with trial information noted in Table 1. However, there remains considerable potential for the validation of the yet clinically untested drugs as potential VS and meningioma treatments, as well as their potential impact on the immune microenvironment.

**Table 1:** FDA/NICE-approved drug repurposing candidates identified in IPA

FDA/NICE drug	Additional names	LINCS target(s)	IPA z-score		Clinical trials		
			VS	Meningioma	*NF2*-SWN	VS	Meningioma
Crizotinib	PF02341066, Xalkori	ALK, ROS1	-51.58	-44.1	**NCT04283669**	**NCT04283669**	None
Bosutinib	SKI-606	RTKs	-50.91	-36.92	None	None	None
Sorafenib	BAY-439006, Nexavar	VEGFR, PDGFR, RAF	-50.83	-46.82	None*	None	None
Mitoxantrone	CL-232325, DHAQ, Novantron, Novantrone	Topo-isomerase	-48.93	-39.64	None	None	None
Nintedanib	BIBF-1120, Vargatef	VEGFR, FGFR, PDGFR	-48.80	-51.58	None	None	None
Lapatinib	GW-572016, Tykerb	HER2, EGFR	-44.55	-46.14	NCT00863122	NCT00973739	None
Everolimus	RAD001, Afinitor	mTOR	-44.51	-39.18	*NCT01345136*, NCT01490476, NCT01880749, NCT01419639	NCT01880749	NCT01880749, **NCT06126588**^#^ NCT02333565^#^ *NCT00972335*^#^
Trametinib	GSK-1120212, JTP-74057	MEK1, MEK2	-43.80	-35.35	None	None	**NCT03631953**^#^ NCT03434262^#^
Sunitinib	Sutent, SU11248	RTKs	-42.30	-37.94	None	None	NCT00589784
Gefitinib	ZD1839, Iressa	EGFR	-40.56	-39.00	None	None	NCT00025675

* Has been in Phase 0 study in *NF2*-SWN peripheral schwannoma EudraCT: 2011–001789-16

Clinical trial status: **Active**,*Terminated*, Completed

^#^ Treatment in combination study

**Fig. 5:**
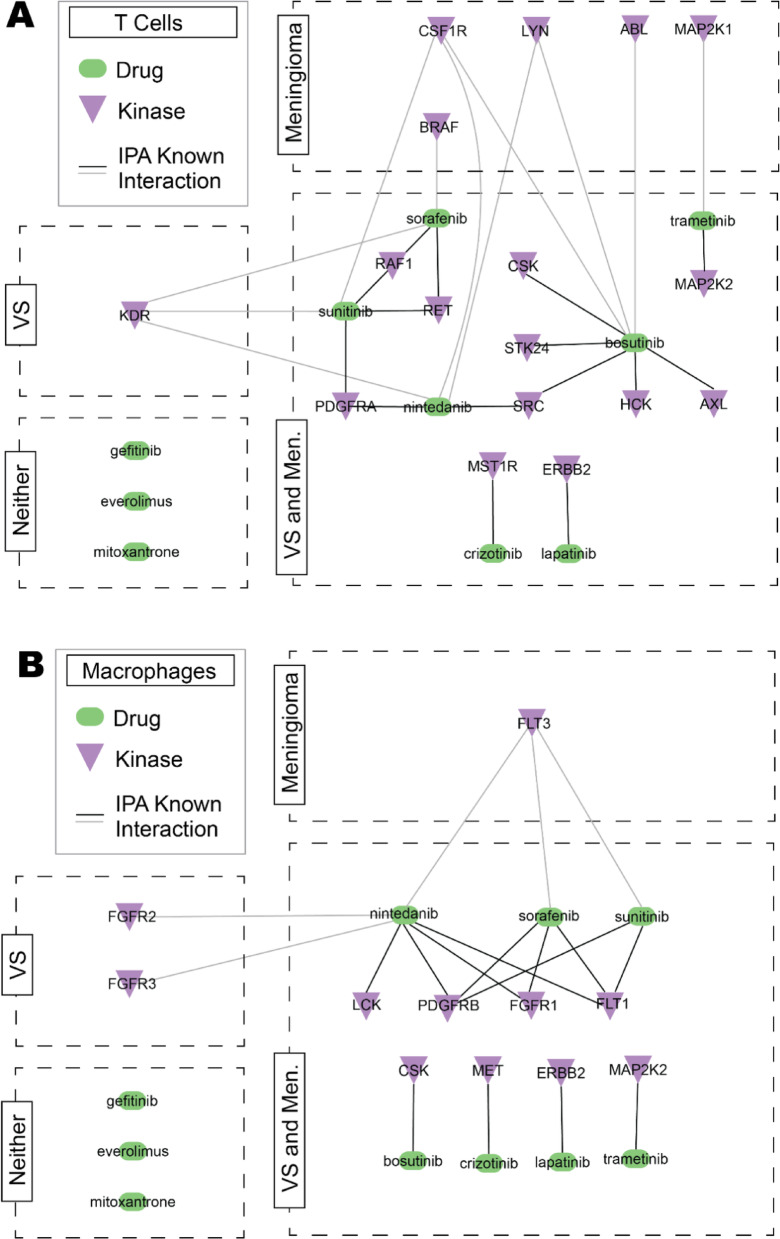
The T cells and macrophages of VS and meningioma share similar drug-target kinases. Pseudo-bulk from single cell RNA sequencing data from T cells and macrophage clusters analysed in Ingenuity Pathway Analysis (IPA). Original data acquired from GSE183655 and GSE216783 from Gene Expression Omnibus containing meningioma (*n* = 6) and VS samples (*n* = 15), respectively. IPA connectivity map of significantly different (Wald test with Benjamini–Hochberg FDR adjustment *p* < 0.05 with fold change ≤ -2 or ≥ 2) and non-significantly different kinases with 10 FDA/NICE approved drugs in **A** T cells and **B** macrophages of VS and meningioma. Abbreviations: Meningioma (Men.), vestibular schwannoma (VS)

The presence and expression of drug-target kinases that were specifically noted to have direct chemical-protein interactions with the 10 FDA/NICE-approved drugs in IPA were investigated. There were 11 kinases that were not significantly differently expressed between meningioma and VS present in the T cells (*RAF1*, *RET*, *PDGFRA*, *SRC*, *STK24*, *CSK*, *MAP2K2*, *HCK*, *AXL*, *MST1R*, and *ERBB2*) (Fig. 5A). Additionally, there were eight kinases that were not significantly differently expressed between meningioma and VS present in the macrophages (*LCK*, *PDGFRB*, *FGFR1*, *FLT1*, *CSK*, *MEK*, *MAP2K2*, and *ERBB2*) (Fig. 5B). Interestingly, three of these drug-target kinases overlapped between T cells and macrophages, namely *MAP2K2* (also named *MEK2*), *ERBB2* (also named *HER2*), and *CSK*. All the kinases presented in Fig. 5 were known drug targets identified for 7/10 of the FDA/NICE-approved drug repurposing candidates noted in Table 1, with the exception of mitoxantrone (which targets topoisomerase and is not known to target kinases), everolimus and gefitinib. As such, despite everolimus being a highly investigated agent in *NF2*-SWN, VS and meningioma tumours, from these analyses in IPA there was no similarity in potential kinase targets in meningioma and VS for everolimus.

Few drug-target kinases were significantly differently expressed between meningioma and VS T cells and macrophages. The T cells in meningioma showed five additional drug-target kinases that were significantly increased in expression compared to VS (*CSF1R*, *LYN*, *ABL*, *MAP2K1*, and *BRAF*), whereas VS had only one drug-target kinase, *KDR* (Fig. 5A). The macrophages in meningioma had one drug-target kinase that was significantly increased in expression compared to VS (*FLT3*), whereas VS had two drug-target kinases, *FGFR2* and *FGFR3* (Fig. 5B). As such, the overall strong similarities of the expression of these drug-target kinases within the T cells and macrophages of meningioma and VS types may implicate kinases as potential targets to modulate the immune tumour microenvironments of both tumour types.

## Discussion

The aim of this study was to address similarities and differences between meningioma and VS to aid with therapeutic repurposing for *NF2*-SWN patients who have synchronous tumour burden. To address this, bulk and single cell gene expression data were explored with a particular focus on the immune compartment. Despite having different tumourigenic cell origins (Schwann or meninge-derived cells), the tumour microenvironments of VS and meningioma presented similarities in the broad types of immune cells present. However, VS were composed of a proportionally larger immune compartment than meningioma and were found to contain more alternatively activated TAMs. Nevertheless, the alternatively and classically activated TAMs in VS had significant signalling pathway suppression compared to meningioma. Interestingly, the activity of the significant pathways in the specific T cell subtypes differed in the two tumour types; CD4 + T cells displayed increased activity, however CD8 + T cells were predicted to be suppressed in VS compared to meningioma. Despite differences in their immune compartments, similarities were identified in the kinases present in the immune cells of meningioma and VS. Overall, these data indicate key similarities and differences in the tumour microenvironments of meningioma and VS which may shed light on potential common therapeutic avenues to reduce tumour growth.

As CD8 + T cells were predicted to be more active in meningioma than VS, this could mean meningioma are more amenable to T cell targeted immunotherapy. Clinical trials are currently underway for checkpoint inhibitors in meningioma patients (nivolumab and ipilimumab; NCT02648997, NCT03279692, and NCT03173950, pembrolizumab NCT03016091, avelumab NCT03267836) which include targeting the PD-1/PD-L1 axis previously suggested for VS [20, 45, 46]. Interestingly, there has been one case to date of a VS showing reduced growth rate after treatment with anti-PD-1 antibody pembrolizumab indicating that benefits from immune checkpoint inhibition could be found for both VS and meningioma [24].

However, as noted by the smaller relative abundance, potential mitochondrial dysfunction, and differences in signalling pathway activity of macrophages in meningioma, it is possible that immune cells may have more of an active role in promoting faster tumour growth in VS compared to meningioma. Meningioma tumours with the highest level of immune infiltration (referred to as the ‘immunogenic’ subtype) have been shown to comprise WHO grade 1 and 2 tumours, with more proliferative ‘tumourigenic’ subtypes displaying more clinically aggressive characteristics [32]. In contrast, a greater macrophage density has repeatedly correlated with faster VS growth in histological, imaging and high dimensional imaging mass cytometry single cell studies [13, 19, 25, 26]. Therefore, it may be necessary to use anti-tumourigenic and immune targeted drug combinations to have a maximal effect on both meningioma and VS. However, as serial MRIs were not available alongside the datasets used in this study, stratification by tumour growth rate was not possible and therefore further work is required to understand the relative influence of specific immune populations over the course of meningioma and VS development.

VS and meningioma share potential drug repurposing candidates that have the potential to modulate the immune microenvironment. However, it is important to note that the drug repurposing in this study was conducted on sporadic VS and meningioma without available information on potential *NF2* gene alterations—a key limitation when assessing its specificity to *NF2*-SWN. Despite this, ten drug candidates were predicted to be effective for both tumour types: crizotinib, bosutinib, sorafenib, mitoxantrone, nintedanib, lapatinib, everolimus, trametinib, sunitinib, and gefitinib. These therapies are already FDA/NICE approved for a variety of tumours including lung cancer, leukaemia, carcinoma, prostate cancer, breast cancer, melanoma, and neuroendocrine tumours. Despite not being approved for meningioma or VS, 6/10 of these drugs have been or are currently being assessed in clinical trials for these tumours (Table 1). As an example, a phase 0 trial by Ammoun et al. (2019) of five *NF2*-SWN patients receiving the maximum tolerable dose of sorafenib for 11 days saw an increase in macrophages and T cells in biopsied peripheral schwannoma [1]. Although the short-term primary outcome of target inhibition was negative, this demonstrates that sorafenib has the potential to modulate the immune microenvironment of *NF2*-SWN-related tumours as suggested in the present study which may alter the growth rate of these tumours over longer treatment windows.

In addition to the six aforementioned drugs, this study identified four potentially viable therapeutics that remain clinically untested that could be assessed in vitro or in vivo before clinical trials: bosutinib, sorafenib, mitoxantrone, and nintedanib. Interestingly, 9/10 drugs target kinases and RTKs, and for 7/10 drugs there were kinase targets present that were not significantly different between meningioma and VS T cells or macrophages, such as *MAP2K2*, *CSK*, and *ERBB2 (HER2* which was also found to be co-overexpressed in both bulk VS and meningioma samples in this study). Therefore, these 7 drugs (excluding gefitinib, mitoxantrone, and everolimus) have the potential to modulate the tumour immune microenvironment in a similar manner in both meningioma and VS through targeting kinase-driven pathways. These treatments may also reduce tumour growth in a multifaceted manner, for example nintedanib may also reduce *COL1A1* expression (found in this study to be co-overexpressed in meningioma and VS compared to their control tissues), and reduce collagen secretion and fibril assembly (thus impacting fibrosis-related pathways also found to be dysregulated in VS in this study) [22].

However, it should be noted that there has been a lack of translation of the 10 identified compounds to the clinic which may indicate several limitations to repurposing these compounds, for example; a long time to clinical translation is required, the targets are less effective in VS and meningioma than the approved conditions, the LINCS database is limited to small molecules and so prevented many antibody-based compounds from assessment in the present analysis, and differences in the tumour microenvironments between VS and meningioma may impact how effective these compounds are for slowing tumour growth. Therefore, although drug repurposing offers the potential of clinical fast-tracking to current patients, novel compounds specifically active toward VS and meningioma immune targets may be required for maximal patient benefit in the future.

The lack of significant difference in the abundance of endothelial cells between meningioma and VS is of interest due to the clinical difference noted in the efficacy of the Vascular Endothelial Growth Factor (VEGF)-targeted monoclonal antibody bevacizumab that is commonly used to treat *NF2*-SWN VS but ineffective for meningioma [17, 27, 33, 41]. Despite not having dual efficacy for VS and meningioma in these patients, the ability to avoid contraindications when taking medications in patients presenting with multiple tumour types is important and bevacizumab does not appear to have adverse effects on meningioma, instead resulting in stable disease [27, 33, 41]. Interestingly in the present study, *IL1B* was more highly expressed in VS than meningioma and therefore drugs such as anakinra and canakinumab, previously suggested as a potential treatment for VS-induced sensorineural hearing loss, [16] may be effective for hearing loss and VS treatment without contraindications in meningioma.

Although this study mainly assessed sporadic tumours, VS from sporadic and *NF2*-SWN patients have previously been shown to have highly similar tumour immune microenvironments showing strong similarities in signalling pathways, gene expression, cell type abundance and imaging mass cytometry staining [15, 25], further reinforced in this study (Supplementary Fig. 4). Interestingly, the majority of sporadic VS harbour *NF2* pathogenic variants which are also common in non-skull base sporadic meningioma [9, 52]. In this study, the GSE216783 dataset contained 12/15 (80%) VS samples with known *NF2* pathogenic variants, and although the *NF2* status of the sporadic meningioma from GSE183655 were unavailable, 3/6 (50%) of these were confirmed to have a loss of 22q (the region in which the *NF2* gene is located) (Supplementary Table 1). Additionally, 4/6 (67%) of the sporadic meningioma were non-skull base (*n* = 3 convexity, and *n* = 1 parasagittal) and therefore were more likely to harbour *NF2* pathogenic variants than the two skull-base meningioma (*n* = 2 middle or posterior cranial fossa) [4, 10, 52]. However, the similarities and differences between meningioma from sporadic and *NF2*-SWN patients have been debated. Although once deemed more aggressive in *NF2*-SWN, [2] more recent assessment of meningioma from sporadic and *NF2*-SWN patients have shown these tumours to be similar in histology, WHO grade, and natural history [12]. In a study of *n* = 14 *NF2*-SWN meningioma and *n* = 15 sporadic *NF2*-altered meningioma, Teranishi et al. (2023) showed similarly low *NF2* gene expression, no distinct separation by PCA, and no significant difference between *NF2*-SWN and *NF2*-altered sporadic meningioma in immune infiltration score by CIBERSORTx [47]. However, Teranishi et al. (2023) also noted *NF2*-SWN meningioma were of a more ‘immunogenic’ subtype than sporadic meningioma, finding that *NF2*-SWN meningioma had greater CD45 + staining by immunohistochemistry, higher *PTPRC* gene expression by bulk RNA-seq, and more myeloid cells in *NF2*-SWN meningioma when deconvolved by xCell [47]. As such, although there are similarities between *NF2*-SWN and sporadic tumours, the present study is limited by the predominant use of sporadic samples due to the lack of publicly available datasets containing both *NF2*-SWN VS and *NF2*-SWN meningioma. Therefore, a study comprising matched meningioma and VS from the same *NF2*-SWN patients would be valuable in providing a more in-depth and high dimensional spatial interrogation of these two tumour types in the target patient group.

## Concluding remarks

This study assessed meningioma and VS for their similarities and differences focussing on the immune compartment of the two tumour types. While meningioma and VS shared overall similarities in their broad immune microenvironments, differences in the relative abundance of their immune compartments and the predicted activity of their immune subtypes may mean these tumours have varying success with potential immunotherapeutics. However, rapid clinical translation by repurposing FDA/NICE approved drugs such as bosutinib, sorafenib, mitoxantrone, and nintedanib (which are yet to be clinically assessed in VS or meningioma) in combination with immunotherapies may offer a dual benefit to *NF2*-SWN patients presenting with both meningioma and VS.

## Supplementary Information

Below is the link to the electronic supplementary material.

Supplementary Material 1

## Data Availability

The datasets analysed during the current study are publicly available at the Gene Expression Omnibus repositories GSE54934 [https://www.ncbi.nlm.nih.gov/geo/query/acc.cgi?acc=GSE54934], GSE183655 [https://www.ncbi.nlm.nih.gov/geo/query/acc.cgi?acc=GSE183655] and GSE216783 [https://www.ncbi.nlm.nih.gov/geo/query/acc.cgi?acc=GSE216783].
