# Supplementary material for: The tumour immune microenvironment is enriched but suppressed in vestibular schwannoma compared to meningioma: therapeutic implications for NF2-related schwannomatosis
Source: Acta Neuropathol Commun. 2025 Dec 23;13:256. doi: 10.1186/s40478-025-02176-9 (PMC12729190; doi:10.1186/s40478-025-02176-9)
Supplement: Supplementary file 1 — Supplementary Material 1 [file 40478_2025_2176_MOESM1_ESM.pdf]

{SUPPLEMENTARY INFORMATION}

Supplementary Table 1. Patient clinical information.

	GEO Sample ID	Sample Type	Sex	WHO Grade	Location	Age (years)	Meningioma DNA Methylation Group	NF2-SWN Status	Genetic Variant
GSE54934 bulk Affymetrix	GSM1326668	Healthy Meninge	M	N/A	/	/	N/A	Sporadic	/
	GSM1326669	Healthy Meninge	M	N/A	/	/	N/A	Sporadic	/
	GSM1326670	Healthy Meninge	F	N/A	/	/	N/A	Sporadic	/
	GSM1326671	Meningioma	/	1	/	/	/	Sporadic	/
	GSM1326672	Meningioma	/	2	/	/	/	Sporadic	/
	GSM13266731	Meningioma	/	1	/	/	/	Sporadic	/
	GSM1326674	Meningioma	/	1	/	/	/	*NF2*-SWN patient	/
	GSM1326675	Meningioma	/	1	/	/	/	Sporadic	/
	GSM1326676	Meningioma	/	1	/	/	/	Sporadic	/
	GSM1326677	Meningioma	/	1	/	/	/	Sporadic	/
	GSM1326678	Meningioma	/	1	/	/	/	*NF2*-SWN patient	/
	GSM1326679	Meningioma	/	2	/	/	/	Sporadic	/
	GSM1326680	Meningioma	/	1	/	/	/	Sporadic	/
	GSM1326681	Meningioma	/	1	/	/	/	Sporadic	/
	GSM1326682	Meningioma	/	1	/	/	/	Sporadic	/
	GSM1326683	Meningioma	/	1	/	/	/	Sporadic	/
	GSM1326684	Meningioma	/	1	/	/	/	Sporadic	/
	GSM1326685	Meningioma	/	1	/	/	/	Sporadic	/
	GSM1326686	Meningioma	/	1	/	/	/	Sporadic	/
	GSM1326687	Meningioma	/	1	/	/	/	Sporadic	/
	GSM1326688	Meningioma	/	1	/	/	/	Sporadic	/
	GSM1326689	Meningioma	/	1	/	/	/	Sporadic	/
	GSM1326690	Meningioma	/	1	/	/	/	Sporadic	/
	GSM1326691	Meningioma	/	1	/	/	/	Sporadic	/
	GSM1326692	Meningioma	/	1	/	/	/	Sporadic	/
	GSM1326698	VIII cranial nerve	/	ND	/	/	N/A	Sporadic	/
	GSM1326700	Vestibular Nerve	/	ND	/	/	N/A	Sporadic	/
	GSM1326702	Vestibular Schwannoma	/	ND	/	/	N/A	Sporadic	/
	GSM1326703	Vestibular Schwannoma	/	ND	/	/	N/A	Sporadic	/
	GSM1326704	Vestibular Schwannoma	/	ND	/	/	N/A	Sporadic	/
	GSM1326705	Vestibular Schwannoma	/	ND	/	/	N/A	Sporadic	/
	GSM1326706	Vestibular Schwannoma	/	ND	/	/	N/A	Sporadic	/
	GSM1326707	Vestibular Schwannoma	/	ND	/	/	N/A	Sporadic	/
	GSM1326708	Vestibular Schwannoma	/	ND	/	/	N/A	Sporadic	/
	GSM1326709	Vestibular Schwannoma	/	ND	/	/	N/A	Sporadic	/
	GSM1326711	Vestibular Schwannoma	/	ND	/	/	N/A	Sporadic	/
	GSM1326713	Vestibular Schwannoma	/	ND	/	/	N/A	Sporadic	/
	GSM1326715	Vestibular Schwannoma	/	ND	/	/	N/A	Sporadic	/
	GSM1326717	Vestibular Schwannoma	/	ND	/	/	N/A	Sporadic	/
	GSM1326719	Vestibular Schwannoma	/	ND	/	/	N/A	Sporadic	/
	GSM1326721	Vestibular Schwannoma	/	ND	/	/	N/A	Sporadic	/

	GSM1326723	Vestibular Schwannoma	/	ND	/	/	N/A	Sporadic	/
	GSM1326725	Vestibular Schwannoma	/	ND	/	/	N/A	Sporadic	/
	GSM1326726	Vestibular Schwannoma	/	ND	/	/	N/A	Sporadic	/
	GSM1326728	Vestibular Schwannoma	/	ND	/	/	N/A	Sporadic	/
	GSM1326731	Vestibular Schwannoma	/	ND	/	/	N/A	Sporadic	/
	GSM1326733	Vestibular Schwannoma	/	ND	/	/	N/A	Sporadic	/
	GSM1326735	Vestibular Schwannoma	/	ND	/	/	N/A	Sporadic	/
	GSM1326737	Vestibular Schwannoma	/	ND	/	/	N/A	*NF2*-SWN patient	/
	GSM1326739	Vestibular Schwannoma	/	ND	/	/	N/A	Sporadic	/
	GSM1326741	Vestibular Schwannoma	/	ND	/	/	N/A	*NF2*-SWN patient	/
	GSM1326743	Vestibular Schwannoma	/	ND	/	/	N/A	Sporadic	/
	GSM1326745	Vestibular Schwannoma	/	ND	/	/	N/A	Sporadic	/
	GSM1326747	Vestibular Schwannoma	/	ND	/	/	N/A	Sporadic	/
	GSM1326748	Vestibular Schwannoma	/	ND	/	/	N/A	Sporadic	/
	GSM1326751	Vestibular Schwannoma	/	ND	/	/	N/A	Sporadic	/
	GSM1326753	Vestibular Schwannoma	/	ND	/	/	N/A	Sporadic	/
	GSM1326755	Vestibular Schwannoma	/	ND	/	/	N/A	*NF2*-SWN patient	/
GSE183655 single cell RNA-seq	GSM5567093	Meningioma	F	2	Tentorium/ MCF	44	Merlin intact	Sporadic	22q Intact
	GSM5567094	Meningioma	M	1	Convexity	74	Immune enriched	Sporadic	22q Loss
	GSM5567095	Meningioma	M	2	Parasagittal	59	Hypermitotic	Sporadic	22q Loss
	GSM5567096	Meningioma	F	2	MCF/ PCF	38	Merlin intact	Sporadic	22q Intact
	GSM5567098	Meningioma	M	1	Convexity	86	Immune enriched	Sporadic	22q Loss
	GSM5567101	Meningioma	F	3	Convexity	71	Immune enriched	Sporadic	22q Intact
GSE216783 single cell RNA-seq	GSM6692760	Vestibular Schwannoma	M	ND	/	62	N/A	Sporadic	*NF2* Missense
	GSM6692761	Vestibular Schwannoma	F	ND	/	88	N/A	Sporadic	Unknown
	GSM6692762	Vestibular Schwannoma	F	ND	/	44	N/A	Sporadic	*NF2* Frameshift
	GSM6692763	Vestibular Schwannoma	F	ND	/	59	N/A	Sporadic	*NF2* Frameshift
	GSM6692764	Vestibular Schwannoma	M	ND	/	65	N/A	Sporadic	*NF2* Frameshift
	GSM6692765	Vestibular Schwannoma	M	ND	/	43	N/A	Sporadic	*NF2* Nonsense/ Frameshift
	GSM6692766	Vestibular Schwannoma	F	ND	/	51	N/A	Sporadic	Unknown
	GSM6692767	Vestibular Schwannoma	F	ND	/	64	N/A	Sporadic	*NF2* Nonsense/ Frameshift
	GSM6692768	Vestibular Schwannoma	M	ND	/	72	N/A	Sporadic	Unknown
	GSM6692769	Vestibular Schwannoma	F	ND	/	67	N/A	Sporadic	*NF2* Silent
	GSM6692770	Vestibular Schwannoma	F	ND	/	68	N/A	Sporadic	*NF2* Frameshift
	GSM6692771	Vestibular Schwannoma	M	ND	/	66	N/A	Sporadic	*NF2* Missense/ Frameshift
	GSM6692772	Vestibular Schwannoma	F	ND	/	36	N/A	Sporadic	*NF2* Frameshift
	GSM6692773	Vestibular Schwannoma	M	ND	/	34	N/A	Sporadic	*NF2* Nonsense
	GSM6692774	Vestibular Schwannoma	M	ND	/	37	N/A	Sporadic	*NF2* Indel/ Frameshift

M (Male), F (Female), / (Not Provided), ND (No Data), N/A (Not Applicable), *NF2*-SWN (*NF2*-related schwannomatosis

Supplementary Table 2. Significantly co-over and co-underexpressed DEGs common between VS and meningioma compared to their control tissues.

	VS versus vestibular nerve			Meningioma versus meningeal tissue		
Gene_Symbol	log2FC	P.Value	adj.P.Val	log2FC	P.Value	adj.P.Val
CDH1	3.161173	1.29E-08	2.89E-06	2.466759	0.006056	0.096933
PDGFD	2.926612	1.06E-10	8.09E-08	2.057012	0.003422	0.065064
SLIT2	2.670587	3.97E-11	3.64E-08	1.767518	0.002288	0.048576
SLFN12	1.242644	0.000577	0.010601	1.114551	0.008443	0.117697
ERBB2	1.036888	2.50E-05	0.000986	1.101058	0.000863	0.025211
COL1A2	1.024388	0.009208	0.080278	1.178191	0.005879	0.094998
APLNR	-3.8269	5.77E-11	4.89E-08	-2.27518	5.79E-07	0.000137
AQP4	-3.6134	1.46E-05	0.000656	-3.93948	0.00026	0.010673
SLC14A1	-3.49956	1.14E-07	1.58E-05	-2.05222	4.09E-05	0.002725
BBOX1	-3.37482	8.21E-10	3.55E-07	-1.12878	0.003322	0.063991
AGT	-3.33702	9.22E-14	2.26E-10	-2.3801	7.29E-06	0.000776
ADCYAP1R1	-3.17875	9.88E-10	4.03E-07	-2.40441	9.79E-05	0.005283
MOBP	-3.05281	4.89E-06	0.000293	-2.58718	2.49E-06	0.000374
DCLK1	-3.03732	5.02E-09	1.38E-06	-1.53087	0.003024	0.060141
LGI1	-2.9343	2.80E-08	5.37E-06	-1.58156	0.000634	0.020195
THBS4	-2.87662	7.57E-09	1.94E-06	-1.40654	0.000602	0.019469
HSPB8	-2.85239	3.72E-06	0.000238	-2.7351	0.001915	0.043427
SLAIN1	-2.83046	1.08E-06	9.50E-05	-1.93985	0.000179	0.008263
CXCL2	-2.77279	1.19E-06	0.000102	-1.66953	0.000441	0.015441
ERMN	-2.63994	3.48E-06	0.000228	-1.91086	1.44E-05	0.001249
STMN2	-2.5754	6.76E-05	0.002083	-3.33946	7.51E-08	3.94E-05
TNR	-2.46638	1.82E-06	0.000138	-1.74814	0.002599	0.053191
FABP7	-2.45697	0.000101	0.002869	-2.35553	6.17E-05	0.003699
SCG3	-2.44968	0.000162	0.004138	-3.64398	2.39E-09	3.51E-06
SLCO1C1	-2.44438	1.53E-09	5.61E-07	-1.35829	0.000203	0.009029
TRPM3	-2.41152	8.22E-09	2.06E-06	-1.26548	0.000206	0.009068
PCP4	-2.4005	0.00027	0.005904	-3.64503	5.55E-10	1.13E-06
PHYHIPL	-2.36848	0.000586	0.010712	-2.49617	1.05E-08	1.00E-05
RND1	-2.36017	6.32E-05	0.001978	-2.46985	6.27E-06	0.000688
SPOCK1	-2.35487	7.19E-07	7.01E-05	-1.84917	0.008641	0.119253
SYNPO2	-2.34695	3.38E-06	0.000224	-1.84818	0.0015	0.037349
VSNL1	-2.33935	3.41E-10	1.71E-07	-4.05086	3.38E-07	9.62E-05
GFAP	-2.32035	0.006703	0.063951	-3.47744	0.000427	0.014969
CNTNAP4	-2.31039	1.62E-05	0.000699	-1.99978	0.003342	0.064217
GEM	-2.29127	0.003319	0.038577	-1.30545	0.001592	0.0389
PYGM	-2.26944	5.96E-07	6.14E-05	-1.66104	2.05E-07	7.14E-05
MLC1	-2.24918	6.70E-06	0.000371	-2.0847	1.25E-05	0.001149
STMN4	-2.23257	5.15E-06	0.000304	-1.60337	0.000282	0.011327
SCRG1	-2.20942	0.001567	0.021991	-2.9009	7.88E-06	0.000812
FGFR2	-2.20433	8.93E-06	0.000452	-1.20573	0.003216	0.062831
PNP	-2.19363	8.19E-05	0.002421	-1.14869	0.006365	0.099777
MIR9-1HG	-2.18717	1.14E-05	0.00054	-1.70319	0.005031	0.085022
SLC1A2	-2.18579	1.69E-05	0.000723	-3.21493	1.21E-06	0.000229
LSAMP	-2.18173	0.000605	0.010974	-2.25978	0.006266	0.099141

FAM13C	-2.17518	2.09E-09	7.18E-07	-2.28405	2.64E-10	8.30E-07
DNER	-2.16646	1.95E-05	0.000802	-1.32602	0.003571	0.067216
ETNPPL	-2.15068	0.000832	0.013942	-2.57813	5.16E-08	2.99E-05
CDH20	-2.1304	2.28E-06	0.000163	-1.66046	0.000161	0.007637
RGS4	-2.12987	1.47E-07	1.96E-05	-2.85713	2.18E-07	7.29E-05
SLC7A11	-2.11839	3.43E-05	0.001259	-2.3039	0.003611	0.067521
GNAO1	-2.11175	1.61E-05	0.000698	-3.415	9.07E-09	9.51E-06
IL1RL1	-2.07706	1.69E-06	0.000133	-1.35975	0.005258	0.087857
CNDP1	-2.01211	8.25E-05	0.002434	-1.65712	7.40E-05	0.004272
CA2	-1.98722	0.000357	0.007273	-1.55664	0.001823	0.04211
PCSK1	-1.98447	5.47E-06	0.000318	-1.65689	0.000108	0.005712
RERG	-1.96876	5.33E-09	1.43E-06	-1.33035	0.000406	0.014483
PWAR6	-1.96591	0.000509	0.009647	-2.66224	0.000197	0.008892
SNAP25	-1.96462	3.70E-06	0.000237	-4.27887	7.62E-11	2.80E-07
LRRC32	-1.95803	2.12E-07	2.65E-05	-1.57456	7.69E-06	0.000796
DPYSL5	-1.95177	9.72E-07	8.85E-05	-1.19772	0.000629	0.020119
GPRC5B	-1.93178	3.45E-05	0.001259	-2.16014	0.000232	0.009805
FAM189A2	-1.92658	4.18E-05	0.001438	-1.06437	0.000403	0.014414
FBXO2	-1.92581	0.000209	0.00497	-2.38895	8.58E-09	9.46E-06
ADAMTS4	-1.91587	0.000315	0.006686	-2.22819	2.08E-05	0.001615
HHATL	-1.9145	2.39E-05	0.000953	-1.0531	0.000846	0.024974
SPOCK3	-1.90916	4.18E-05	0.001438	-1.76076	0.001252	0.033035
STON2	-1.89534	8.39E-13	1.32E-09	-1.05498	0.006683	0.101864
S1PR1	-1.88327	8.45E-06	0.000439	-2.1052	3.29E-07	9.53E-05
ACBD7	-1.87256	6.33E-05	0.001978	-1.78779	0.004135	0.073677
CTNND2	-1.87148	0.001064	0.016587	-2.52428	3.85E-06	0.000499
APBA2	-1.84366	2.54E-06	0.00018	-1.27738	0.004771	0.081762
NRIP3	-1.84021	1.78E-06	0.000136	-1.58127	7.61E-06	0.000791
ELAVL3	-1.83737	7.85E-06	0.000414	-2.36671	1.46E-07	5.68E-05
APLP1	-1.83645	1.02E-05	0.000501	-1.39993	0.000259	0.010673
HOMER1	-1.81281	1.23E-05	0.000576	-2.18737	5.39E-06	0.000625
ACTN2	-1.77897	7.16E-06	0.000386	-1.11427	0.000683	0.021321
ADAMTS1	-1.7711	0.000168	0.004234	-2.69063	7.88E-07	0.00017
RGS16	-1.76469	0.003571	0.04057	-1.52707	0.002353	0.049343
DGKB	-1.75142	4.12E-05	0.001429	-3.12096	4.50E-09	5.51E-06
DTNA	-1.75086	0.000273	0.005964	-2.44392	2.76E-05	0.001983
PPFIA2	-1.73645	1.06E-12	1.53E-09	-2.37593	4.50E-07	0.000117
PVALB	-1.7292	0.001047	0.016412	-2.32022	0.00035	0.013056
SERPINI1	-1.72877	0.00017	0.004269	-2.0657	0.000679	0.021321
RANBP3L	-1.72475	0.001382	0.020038	-2.10488	0.009208	0.124177
GRIA4	-1.72422	0.000251	0.005593	-1.86944	2.66E-05	0.00194
PLCXD3	-1.70587	3.10E-07	3.61E-05	-1.6462	1.09E-06	0.000219
MCTP1	-1.70346	7.98E-06	0.000419	-1.82822	1.12E-06	0.000222
RCAN2	-1.68525	0.000403	0.007972	-2.42431	3.00E-08	2.00E-05
CCL19	-1.68364	0.000284	0.006162	-1.49161	0.00041	0.014556
IRAG1	-1.67361	3.04E-06	0.000206	-1.44788	4.60E-07	0.000117
SPOCK2	-1.67161	3.42E-06	0.000226	-1.41018	0.000222	0.009463
CADPS	-1.66485	9.86E-06	0.000488	-2.0151	8.11E-06	0.000828
MAPK4	-1.66256	0.000131	0.003518	-1.77928	0.000415	0.014677

ITGB3	-1.66134	2.11E-06	0.000154	-1.09109	0.006365	0.099777
MAP7	-1.65914	2.82E-05	0.001083	-1.19004	0.000213	0.009261
ERRFI1	-1.6411	0.000173	0.004333	-1.15882	0.007955	0.113238
GABRB1	-1.6312	1.11E-05	0.000532	-2.13224	6.32E-07	0.000145
MAPK10	-1.5988	1.35E-05	0.000617	-1.45221	0.000216	0.009326
RNF152	-1.58632	8.58E-08	1.30E-05	-1.34357	0.005589	0.091883
RAB3C	-1.58053	6.44E-09	1.69E-06	-2.24867	3.59E-06	0.000485
MYOM1	-1.56685	4.86E-07	5.25E-05	-1.05914	8.44E-07	0.000179
DCLK2	-1.56521	0.00018	0.004464	-1.50257	0.000759	0.022983
STXBP5L	-1.54772	0.000149	0.003858	-2.38632	8.50E-08	3.99E-05
SLC38A3	-1.54673	2.80E-05	0.001079	-1.27654	0.000313	0.011977
PPP2R2C	-1.53416	3.07E-05	0.001155	-1.86565	1.99E-05	0.001563
PHGDH	-1.53133	7.52E-07	7.15E-05	-1.11715	0.000187	0.008545
SLC16A7	-1.52772	5.08E-09	1.38E-06	-1.16731	0.008026	0.113753
SLC25A18	-1.52128	1.04E-05	0.00051	-1.89834	0.001522	0.037766
PDE4B	-1.51381	0.000857	0.014257	-1.05959	0.00092	0.026377
MMRN1	-1.50377	0.00012	0.003286	-1.65426	4.82E-06	0.000587
DGKG	-1.49212	1.73E-05	0.000735	-1.58833	4.61E-06	0.000571
CACNG7	-1.49081	2.28E-06	0.000163	-2.09078	1.33E-06	0.000243
TIMP4	-1.46933	0.000259	0.005705	-1.12589	0.000886	0.025716
BRINP1	-1.4634	4.80E-05	0.001608	-1.45451	0.002337	0.049181
NRG3	-1.46112	0.000895	0.014681	-1.58385	0.000121	0.006214
ATP6V1G2	-1.45581	0.000112	0.002877	-2.02203	1.28E-05	0.001148
PSD2	-1.4504	3.40E-06	0.000225	-1.26746	0.000555	0.018436
DUSP5	-1.43692	0.000325	0.006823	-1.10687	0.002203	0.04739
FLT1	-1.43415	0.000343	0.007127	-1.59178	6.06E-05	0.003673
MYCT1	-1.42708	3.30E-06	0.000222	-1.21739	2.74E-05	0.001974
RNF182	-1.42147	6.61E-05	0.002044	-1.11561	0.001081	0.029609
DLG2	-1.37707	0.00021	0.004981	-1.89735	0.000199	0.0089
RORA	-1.37704	1.54E-05	0.00068	-1.00438	0.001872	0.042703
MYRIP	-1.37063	7.88E-08	1.21E-05	-1.75902	1.81E-08	1.33E-05
TAGLN	-1.35182	0.005137	0.052687	-1.28384	0.002594	0.053125
DNM1	-1.34419	6.45E-07	6.51E-05	-2.49862	4.94E-06	0.000595
MXD1	-1.34305	0.003092	0.036494	-1.43732	0.000105	0.005608
KCNA5	-1.34101	1.22E-05	0.000575	-1.55196	1.23E-08	1.13E-05
CHGB	-1.3302	2.20E-05	0.000889	-1.99107	0.000564	0.018603
ARHGEF4	-1.33005	9.40E-07	8.67E-05	-1.23669	9.29E-06	0.000922
RAMP3	-1.32559	1.10E-07	1.53E-05	-1.06899	8.20E-07	0.000175
PPP1R16B	-1.31469	5.28E-08	8.70E-06	-1.67956	7.10E-08	3.82E-05
PTPRK	-1.30615	0.00042	0.008233	-1.15668	0.003247	0.063183
TAGLN3	-1.3059	0.000116	0.003198	-2.42641	2.11E-07	7.17E-05
ST6GALNAC3	-1.30417	8.33E-08	1.26E-05	-1.17072	0.000199	0.008442
TMEFF2	-1.26826	0.005868	0.057998	-1.98579	0.001132	0.030618
TMEM144	-1.26377	0.008407	0.075254	-1.67606	1.40E-06	0.000252
RUNX1T1	-1.25861	0.000631	0.011335	-1.97253	2.29E-05	0.001732
AIFM3	-1.24596	2.55E-06	0.00018	-1.55835	6.92E-06	0.000745
PDE8B	-1.24502	0.004819	0.050358	-1.21079	0.006126	0.097906
ALDH1L1	-1.24373	4.91E-05	0.001629	-1.05937	0.000633	0.020193
HIVEP2	-1.23486	0.00054	0.010035	-1.54246	5.89E-05	0.003648

TSPAN2	-1.23454	0.002541	0.031476	-1.61955	0.000175	0.008139
SLC39A12	-1.22979	0.000323	0.006805	-1.54596	8.69E-05	0.004839
CAMK2N1	-1.22852	0.000253	0.005621	-1.94768	0.000334	0.012597
TESC	-1.22437	1.30E-08	2.89E-06	-1.34713	0.003254	0.063183
PPP1R1B	-1.21839	4.23E-07	4.68E-05	-1.49423	2.59E-05	0.001901
PRKCB	-1.20334	0.000641	0.011485	-1.83647	5.34E-06	0.000625
SNAP91	-1.20261	5.54E-05	0.001799	-2.42132	1.79E-07	6.82E-05
PCDH19	-1.19883	0.000431	0.008406	-1.08366	0.006457	0.100394
GGT5	-1.19823	0.002159	0.027874	-1.12895	0.000828	0.02452
CHST1	-1.1972	0.000135	0.003595	-1.47366	5.55E-06	0.000631
HPCAL4	-1.19284	3.92E-07	4.36E-05	-1.82665	0.00107	0.029398
HSD17B14	-1.18989	0.009931	0.084569	-1.19263	0.008172	0.115168
EFHD1	-1.18804	0.000893	0.01467	-1.19859	0.000281	0.011327
GPC5	-1.1861	5.28E-05	0.001735	-1.88595	4.32E-07	0.000115
THRB	-1.17906	3.86E-06	0.000244	-2.11008	1.15E-07	4.96E-05
NCAN	-1.17069	0.000211	0.004985	-1.3303	0.003918	0.070944
HTR2A	-1.16079	1.16E-05	0.000547	-1.97252	0.000777	0.023353
TIE1	-1.15645	1.45E-05	0.000653	-1.06108	2.61E-05	0.001911
FSD1L	-1.14598	0.001913	0.025523	-1.22282	5.49E-07	0.000136
APC	-1.14576	0.002698	0.032981	-1.22169	0.009008	0.122624
SH3GL2	-1.12982	0.000247	0.005532	-1.45422	0.000183	0.008427
SLC7A1	-1.12748	0.001075	0.016667	-1.47582	0.008666	0.119316
ASTN1	-1.12305	7.04E-05	0.002153	-1.18306	0.004633	0.080128
BRINP3	-1.10194	0.000163	0.004159	-1.14699	0.000592	0.019266
RAB6B	-1.10027	0.000145	0.003769	-1.39394	0.000206	0.009068
SYT1	-1.09981	8.83E-05	0.00256	-3.24368	6.96E-05	0.00405
MRTFB	-1.09554	4.14E-08	7.37E-06	-1.13951	0.001646	0.03956
ICAM2	-1.09489	1.26E-05	0.000584	-1.17488	0.000911	0.026181
ADAMTS9	-1.09352	0.00443	0.047392	-1.8801	0.001495	0.037309
KALRN	-1.08546	7.30E-06	0.000391	-1.20633	0.004724	0.081311
SNORD64	-1.06089	0.001083	0.016745	-1.37967	0.000457	0.015895
PTP4A3	-1.04863	0.001127	0.017138	-1.94325	3.55E-06	0.000483
CPNE6	-1.03836	6.18E-05	0.001942	-1.6308	9.06E-06	0.000903
CX3CL1	-1.02639	6.04E-07	6.19E-05	-1.5664	2.07E-09	3.51E-06
PHACTR3	-1.02471	1.82E-06	0.000138	-1.39398	4.09E-06	0.000524
KLHL32	-1.00897	0.008882	0.078084	-1.13122	0.001078	0.02959
CNTFR	-1.0046	0.000117	0.003228	-1.73058	0.002168	0.047117
ARFGEF3	-1.00268	0.003496	0.039918	-1.41269	0.000306	0.011831
CCDC85A	-1.0019	5.29E-05	0.001735	-1.06699	2.34E-05	0.001752

Supplementary Table 3. Top 5 ranked IPA pathway categories and significant pathways from co-over and co-underexpressed DEGs common between VS and meningioma compared to their control tissues.

Top 5 Pathway Categories	Significant Pathways	-log10(Pval)	Ratio	IPA z-score
1. Cellular Stress and Injury	Apelin Liver Signaling Pathway	4.44	0.2	-1
	Hepatic Fibrosis Signaling Pathway	2.7	0.0272	-1.667
	Apelin Cardiomyocyte Signaling Pathway	1.82	0.0408	-2
2. Neurotransmitters and Other Nervous System Signaling	Glutaminergic Receptor Signaling Pathway	4.22	0.038	-2.887
	Neuregulin Signaling	3.07	0.0526	-0.447
	Semaphorin Neuronal Repulsive Signaling	2.56	0.0417	0
	Axonal Guidance Signaling	2.52	0.0244	Unknown
	Serotonin Receptor Signaling	1.9	0.0222	-2.53
	ERBB Signaling	1.9	0.043	Unknown
	Endocannabinoid Neuronal Synapse	1.84	0.0338	-2.236
	Glutamate Receptor Signaling	1.62	0.0469	Unknown
	CDK5 Signaling	1.61	0.0351	0
	Agrin Interactions at Neuromuscular Junction	1.55	0.0441	Unknown
	Melatonin Signaling	1.53	0.0435	Unknown
	CREB Signaling in Neurons	1.53	0.0185	-3.162
	Synaptogenesis Signaling Pathway	1.52	0.0227	-1.89
	Endocannabinoid Developing Neuron	1.46	0.0315	-1
	Synaptic Long Term Depression	1.43	0.0263	-1.342
	GABA Receptor Signaling	1.43	0.0308	Unknown
	Adrenergic Receptor Signaling Pathway	1.42	0.0262	-2.236
	GABAergic Receptor Signaling Pathway	1.35	0.029	-1
3. Neuronal System	Neurotransmitter release cycle	3.23	0.1	-2
	Neurexins and neuroligins	2.65	0.0702	-2
	GABA synthesis, release, reuptake and degradation	1.85	0.105	Unknown
	Glutamate binding, activation of AMPA receptors and synaptic plasticity	1.44	0.0645	Unknown
	Neurotransmitter uptake and metabolism in glial cells	1.42	0.25	Unknown
	Activation of NMDA receptors and postsynaptic events	1.33	0.0361	Unknown
4. Growth Factor Signaling	Neuregulin Signaling	3.07	0.0526	-0.447
	ERBB Signaling	1.9	0.043	Unknown
	Endocannabinoid Developing Neuron	1.46	0.0315	-1
	Regulation of the Epithelial Mesenchymal Transition by Growth Factors Pathway	1.44	0.0266	-0.447
5. Cellular Growth, Proliferation and Development	Hepatic Fibrosis Signaling Pathway	2.7	0.0272	-1.667
	FAK Signaling	2.48	0.0222	-2.673
	Remodeling of Epithelial Adherens Junctions	2.42	0.0606	Unknown
	ILK Signaling	1.92	0.0306	-0.447
	CREB Signaling in Neurons	1.53	0.0185	-3.162
	STAT3 Pathway	1.38	0.0296	Unknown
	BEX2 Signaling Pathway	1.37	0.0375	Unknown
	DHCR24 Signaling Pathway	1.36	0.0292	-1

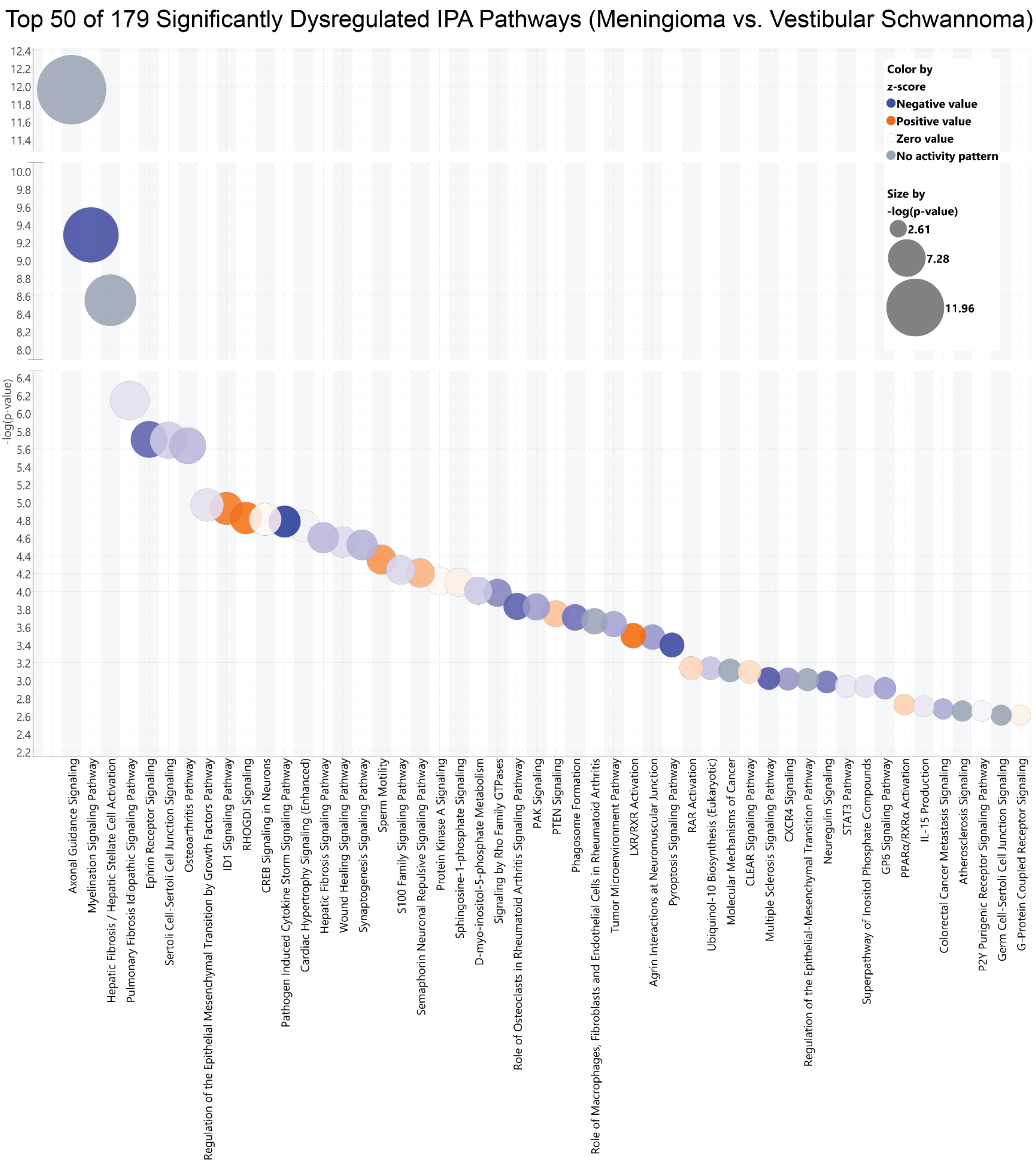

Supplementary Fig 1. Top 50 of the 179 IPA pathways differently significantly dysregulated between meningioma and VS directly. Data acquired from GSE54934 from Gene Expression Omnibus, analysed in Ingenuity Pathway Analysis. Samples included VS (sporadic *n*=28 and NF2 *n*=3), and meningioma (sporadic *n*=20 and NF2 *n*=2).

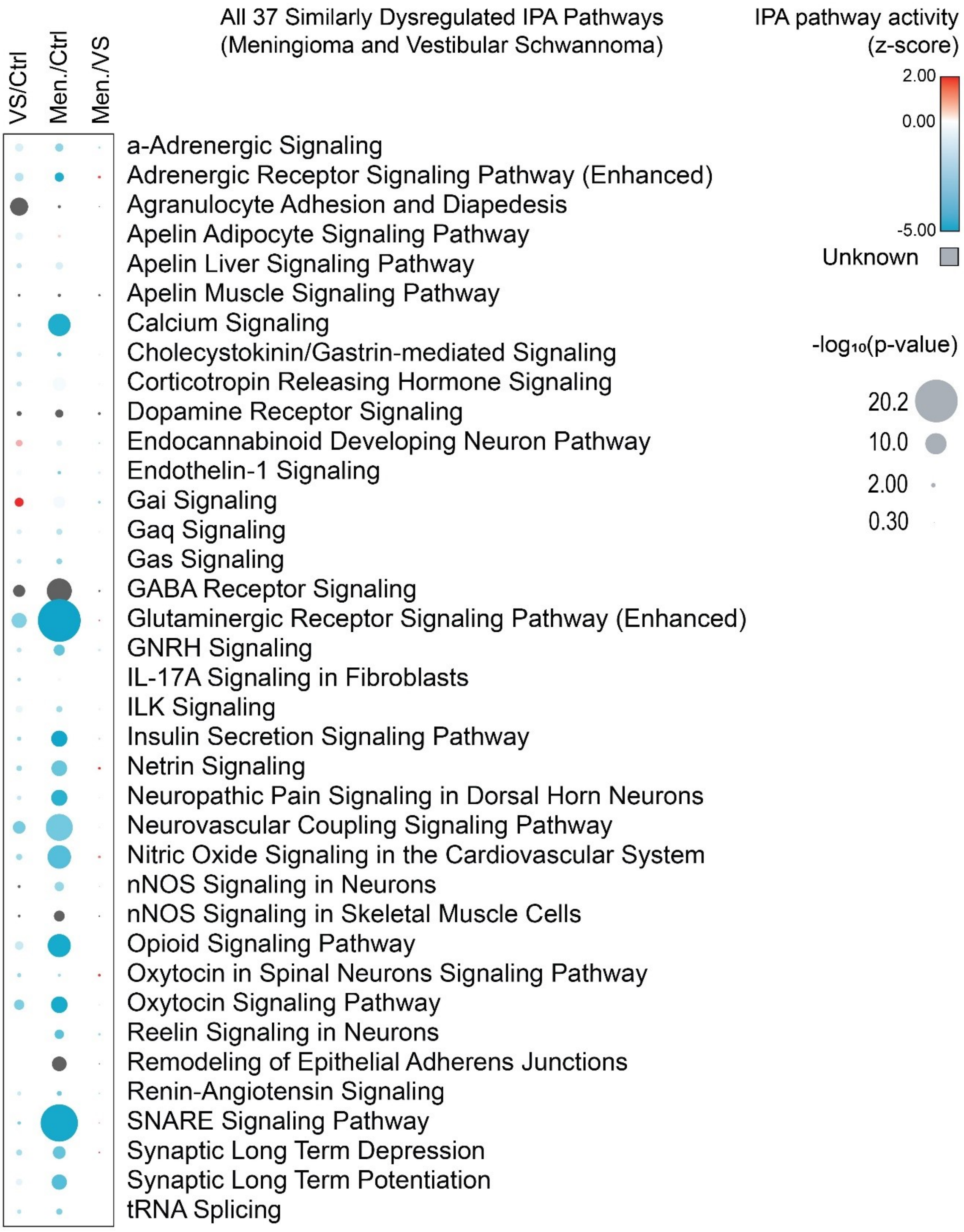

Supplementary Fig 2. 37 IPA pathways similarly significantly dysregulated between VS/control and meningioma/control, that were not significantly different between VS and meningioma directly. Data acquired from GSE54934 from Gene Expression Omnibus, analysed in Ingenuity Pathway Analysis. Samples included VS (sporadic *n*=28 and NF2 *n*=3), control vestibular nerve (*n*=2), meningioma (sporadic *n*=20 and NF2 *n*=2), and control meningeal tissue (*n*=3).

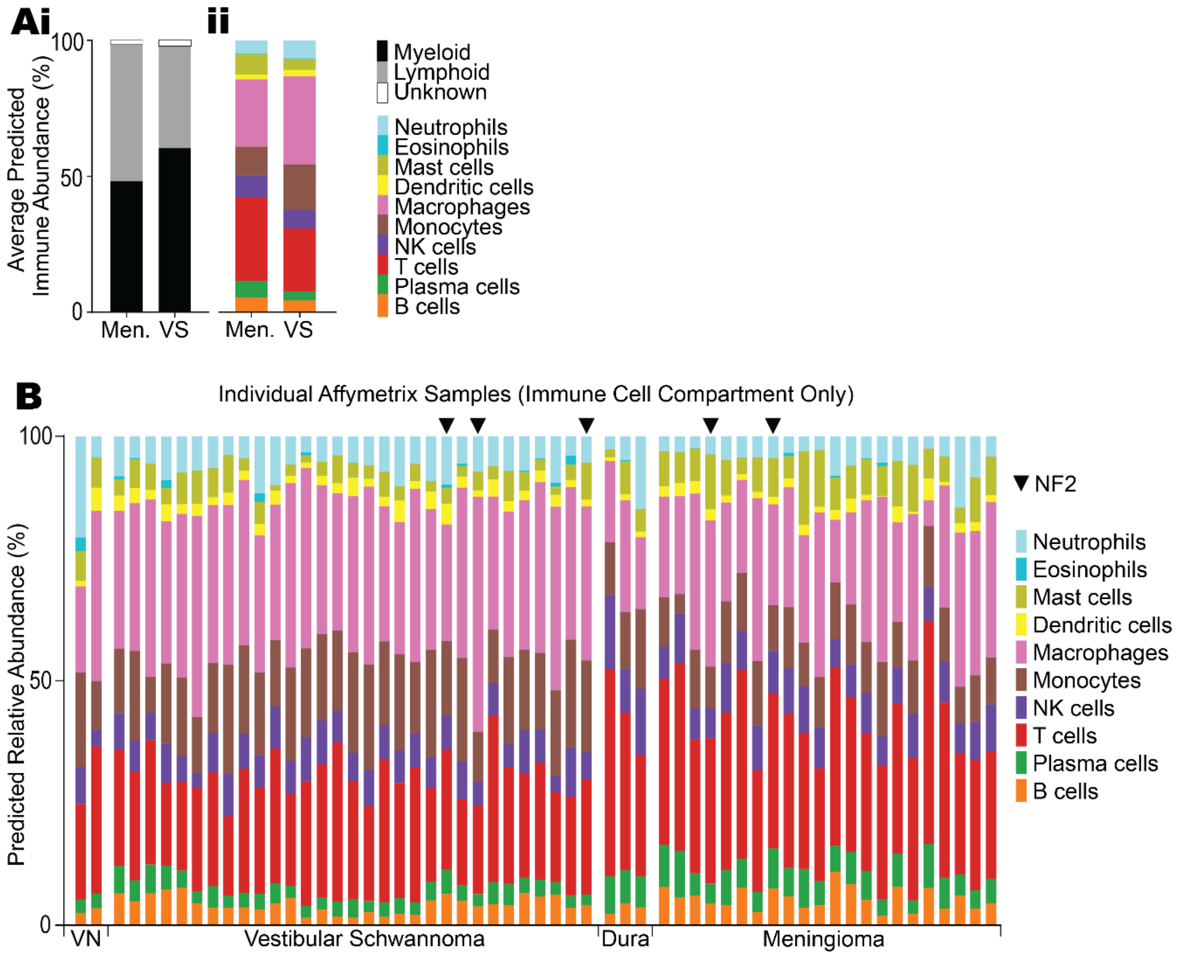

Supplementary Fig 3. Predicted relative abundance of cells in individual Affymetrix samples (immune cell compartment only). Bulk expression data acquired from GSE54934 from Gene Expression Omnibus containing VS (sporadic *n*=28 and NF2 *n*=3), control vestibular nerve (VN, *n*=2), meningioma (sporadic *n*=20 and NF2 *n*=2), and control meningeal dura tissue (*n*=3). **(A)** CIBERSORTx deconvolution into averaged predicted abundance of immune cell origin (Ai) and specific cell types (Aii). **(B)** CIBERSORTx deconvolution by individual sample.

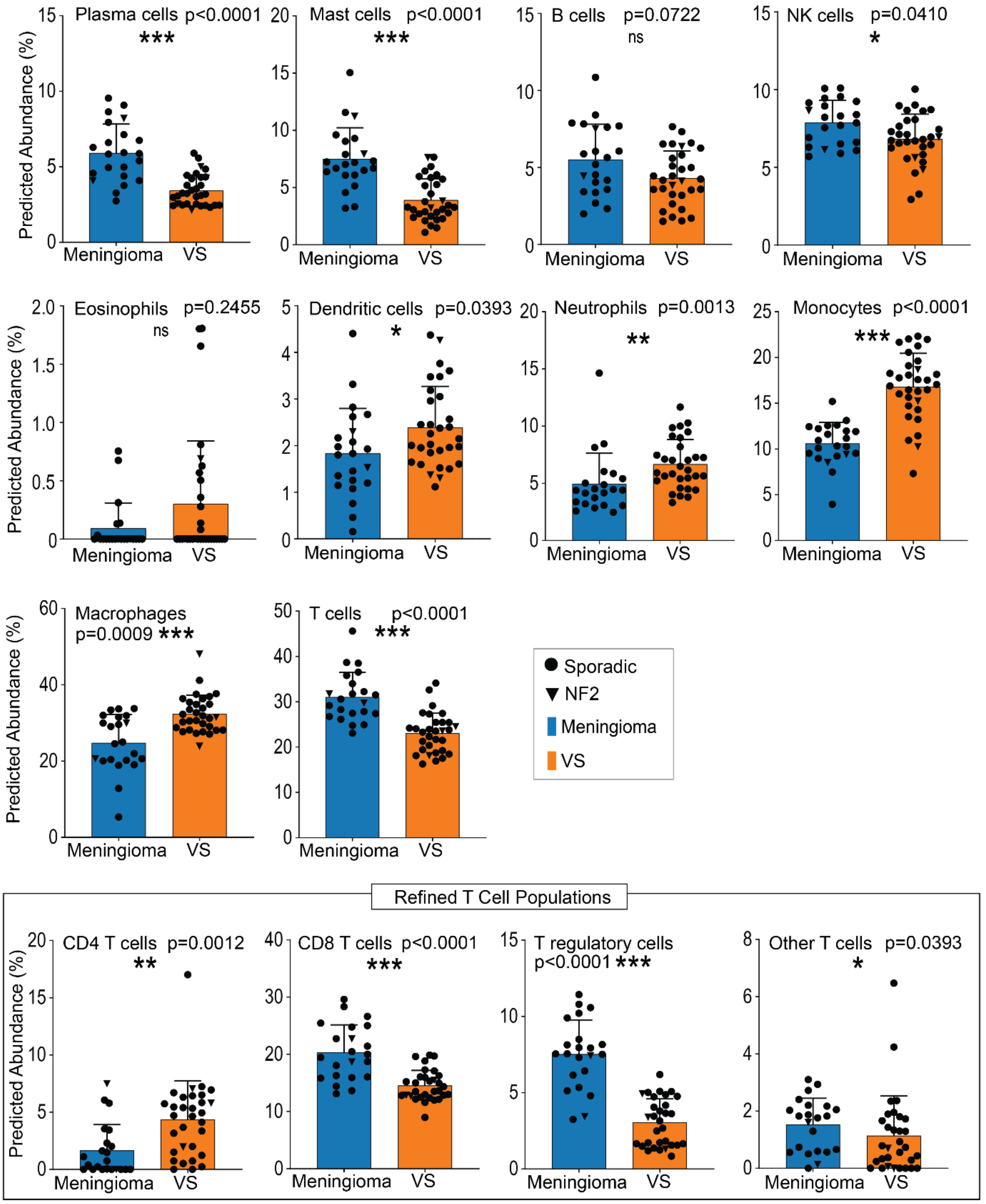

Supplementary Fig 4. VS contain more myeloid cells compared to meningioma which are more lymphoid dominated. Bulk expression data acquired from GSE54934 from Gene Expression Omnibus containing VS (sporadic n=28 and NF2 n=3) and meningioma samples (sporadic n=20 and NF2 n=2). The predicted abundance from broad immune cell types and refined T cell subtypes using CIBERSORTx. Shapiro Wilk normality test followed by Mann-Whitney U test with Benjamini-Hochberg adjustment where significance was determined at * $p < 0.05$, ** $p < 0.01$, *** $p < 0.001$. Abbreviations: meningioma (Men.), vestibular schwannoma (VS), not significant (ns), natural killer cells (NK cells).

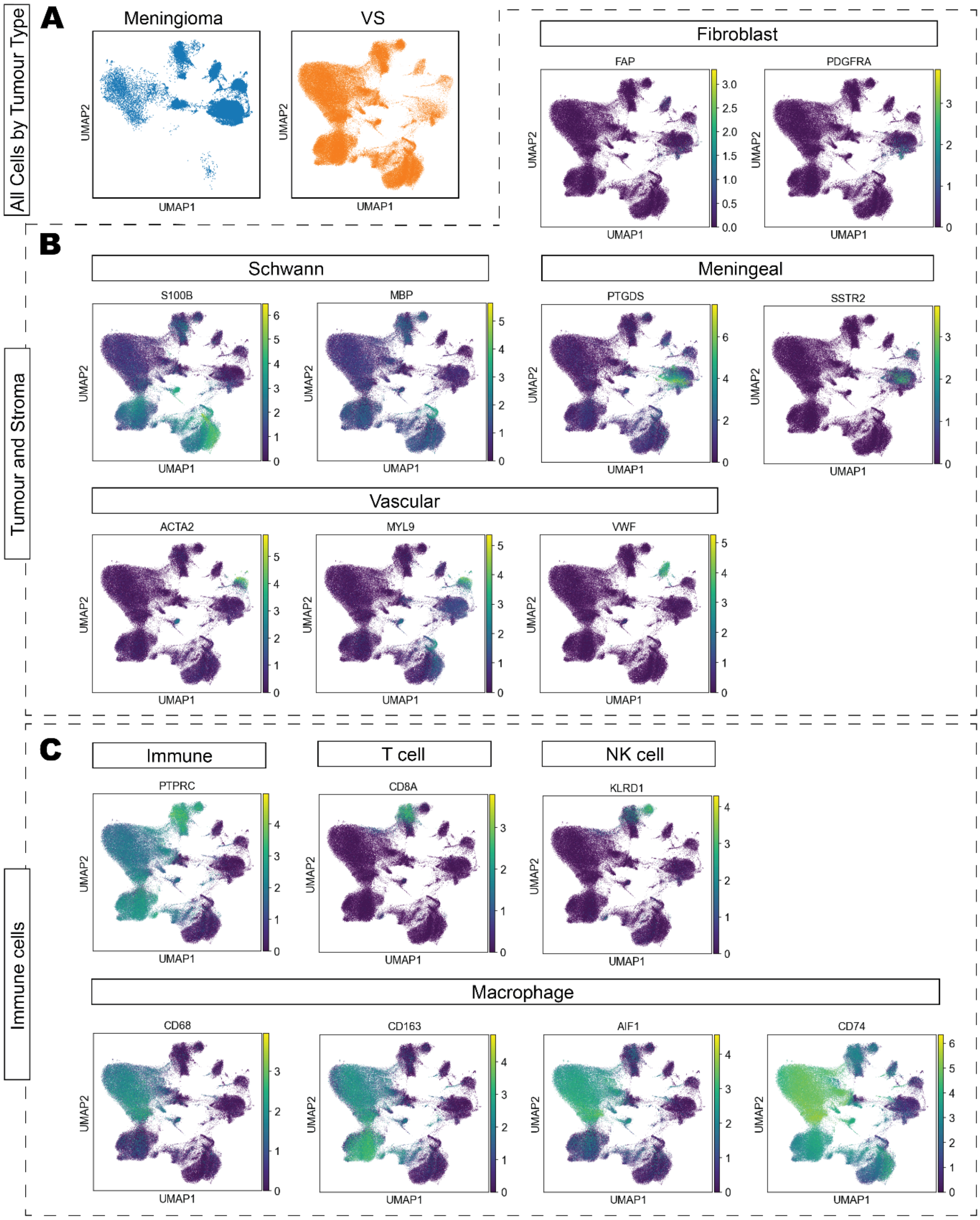

Supplementary Fig 5. Single cell UMAPs. Integrated single cell RNA sequencing data acquired from GSE183655 and GSE216783 from Gene Expression Omnibus containing meningioma (*n*=6) and VS samples (*n*=15), respectively. **(A)** Total cells from each tumour type (meningioma and vestibular schwannoma, VS). **(B-C)** Cell type markers used to validate cell cluster annotations for tumour and stromal cell types (B) and immune cell types (C).

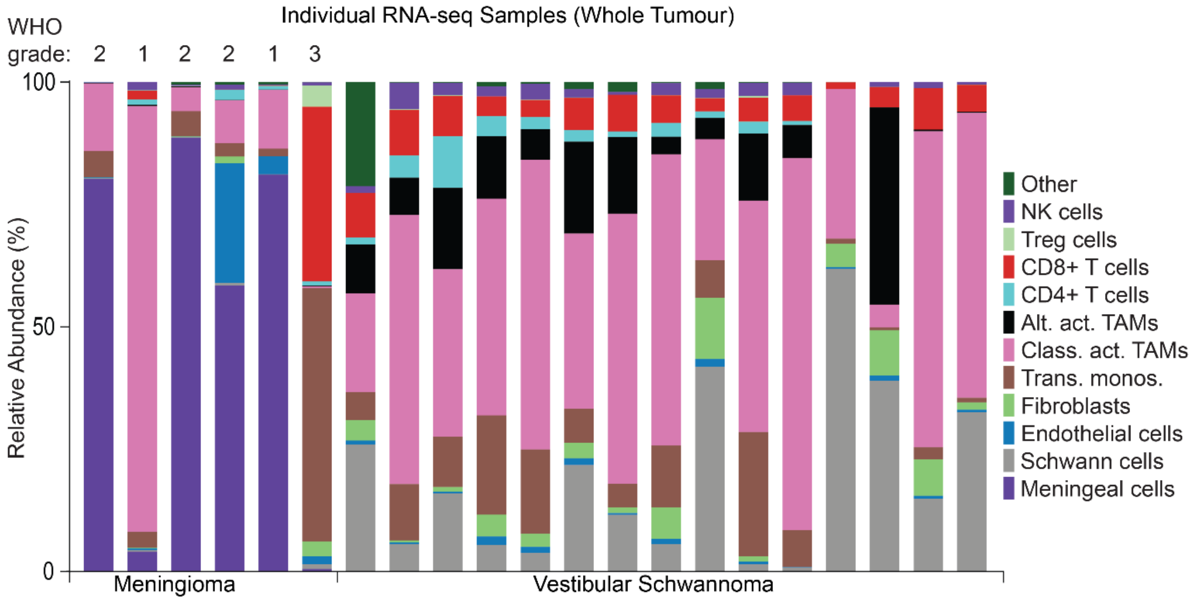

Supplementary Fig 6. Relative abundance of cells in individual RNA-seq samples (whole tumour microenvironment). Relative abundance of cells from whole tumour microenvironment single cell RNA sequencing data acquired from GSE183655 and GSE216783 from Gene Expression Omnibus containing meningioma (*n*=6) and VS samples (*n*=15), respectively.

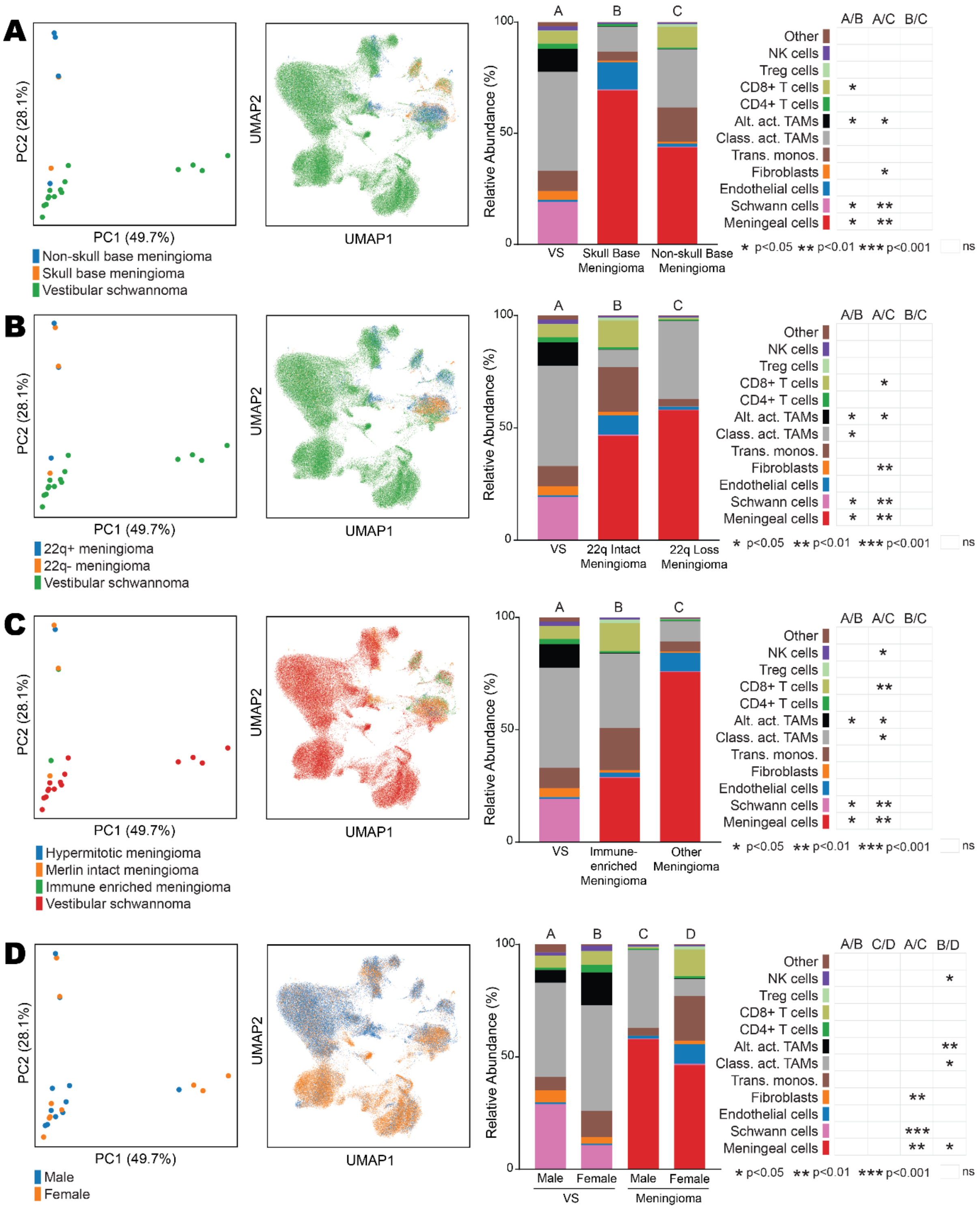

Supplementary Fig 7. Clinical categorisation and comparison of meningioma and vestibular schwannoma (VS) by tumour microenvironment cell types. Single cell RNA sequencing data acquired from GSE183655 and GSE216783 from Gene Expression Omnibus containing meningioma (*n*=6) and VS samples (*n*=15), respectively. Principal component analyses (PCA), Uniform Manifold Approximation Projection (UMAP), and cell type abundance. Data not normally distributed as determined by Shapiro-Wilk test followed by Kruskal-Wallis with significance at p<0.05 (*), p<0.01 (**) and p<0.001 (***). **(A)** Comparisons between VS, skull base and non-skull base meningioma. **(B)** Comparisons between VS, 22q+ and 22q- meningioma. **(C)** Comparisons between VS, immune-enriched and other methylation group meningioma. **(D)** Comparisons between male and female VS and meningioma.

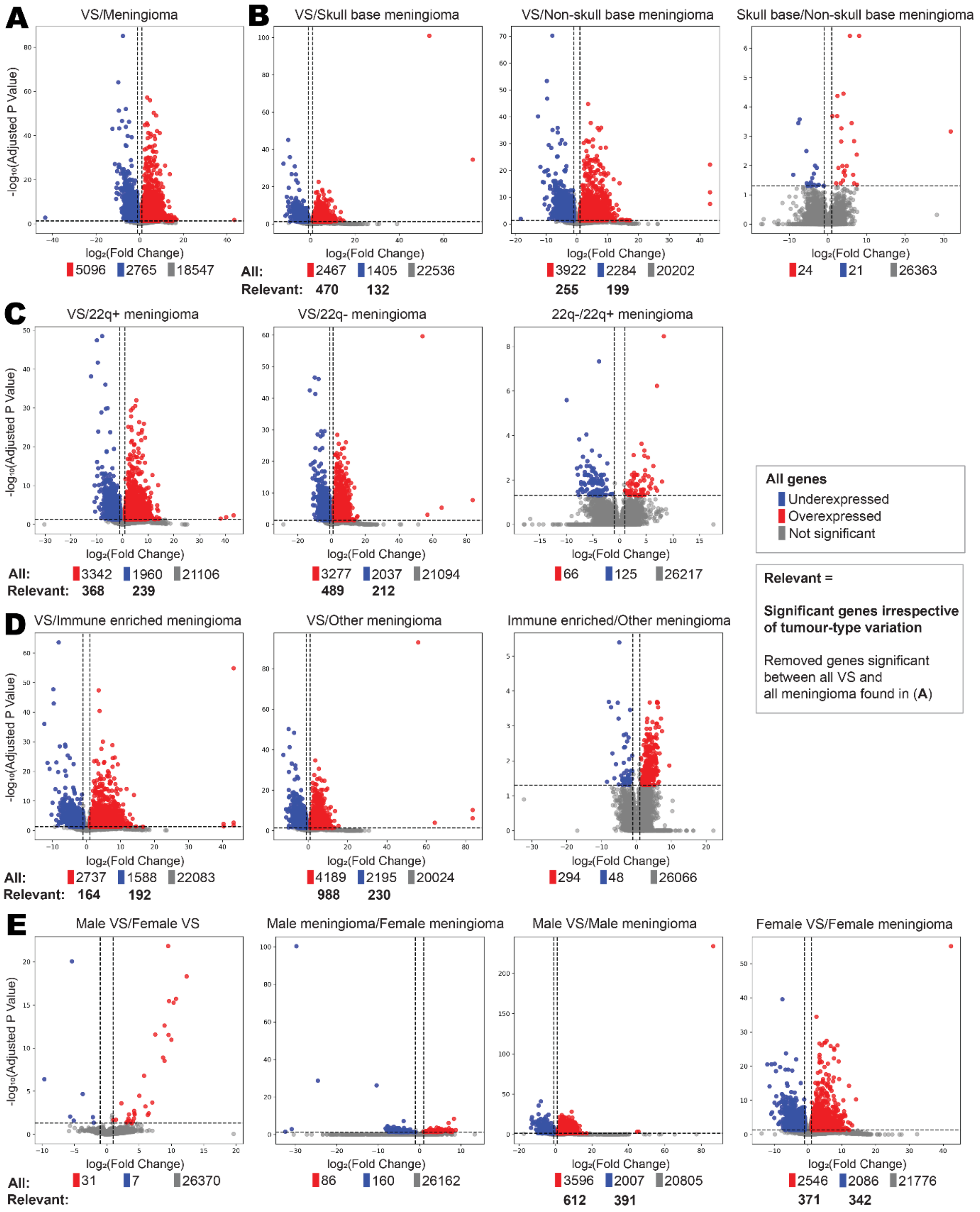

Supplementary Fig 8. Clinical categorisation and comparison of meningioma and vestibular schwannoma (VS) by differential gene expression. Pseudo-bulk from single cell RNA sequencing data from VS and categorised meningioma. **(A)** Comparisons between all VS and all meningioma. **(B)** Comparisons between VS, skull base and non-skull base meningioma. **(C)** Comparisons between VS, 22q+ and 22q- meningioma. **(D)** Comparisons between VS, immune-enriched and other methylation group meningioma. **(E)** Comparisons between male and female VS and meningioma. Differentially expressed genes (DEG) were defined using the Wald test with Benjamini-Hochberg adjustment, significance as $p < 0.05$ and fold change >2 or <2. Original data acquired from GSE183655 and GSE216783 from Gene Expression Omnibus containing meningioma (n=6) and VS samples (n=15), respectively.

Supplementary Table 4. Top 10 ranked IPA significant pathways from relevant DEGs (after accounting for inter-tumoural variation) between VS and immune-enriched meningioma or other methylation group meningioma.

Comparison	Top 10 IPA pathway name	$-\log_{10}$(P value)	Ratio	z-score
Vestibular Schwannoma versus Immune-enriched Meningioma	1. Mitotic Prometaphase	8.08	0.0732	-3.873
	2. Cell Cycle Checkpoints	7.24	0.0588	-4
	3. Kinetochore Metaphase Signaling Pathway	6.67	0.0962	-0.378
	4. Mitotic Metaphase and Anaphase	6.45	0.0593	-3.742
	5. RHO GTPases Activate Formins	5.48	0.0714	-3.162
	6. Nucleotide Excision Repair	4.58	0.0734	-2.121
	7. Cohesin Chromatin Regulation Pathway	4.44	0.0455	-1.667
	8. Mitotic Roles of Polo-Like Kinase	4.04	0.0896	-1.342
	9. FAT10 Cancer Signaling Pathway	3.64	0.098	-1.342
	10. Regulation of mitotic cell cycle	3.39	0.0682	-2.449
Vestibular Schwannoma versus Other Methylation Group Meningioma	1. Pathogen Induced Cytokine Storm Pathway	28.2	0.191	6.197
	2. Th1 and Th2 Activation Pathway	25.9	0.273	Unknown
	3. Neutrophil degranulation	23.4	0.157	8.429
	4. Th1 Pathway	22.5	0.304	5.112
	5. Th2 Pathway	22.4	0.284	3.536
	6. Multiple Sclerosis Signaling Pathway	18.9	0.205	4.323
	7. Macrophage Classical Activation Signaling Pathway	18.3	0.218	3.773
	8. Phagosome Formation	16.6	0.115	6.975
	9. Macrophage Alternative Activation Pathway	16.3	0.203	4.7
	10. Tuberculosis Active Signaling Pathway	15.4	0.176	-2.469

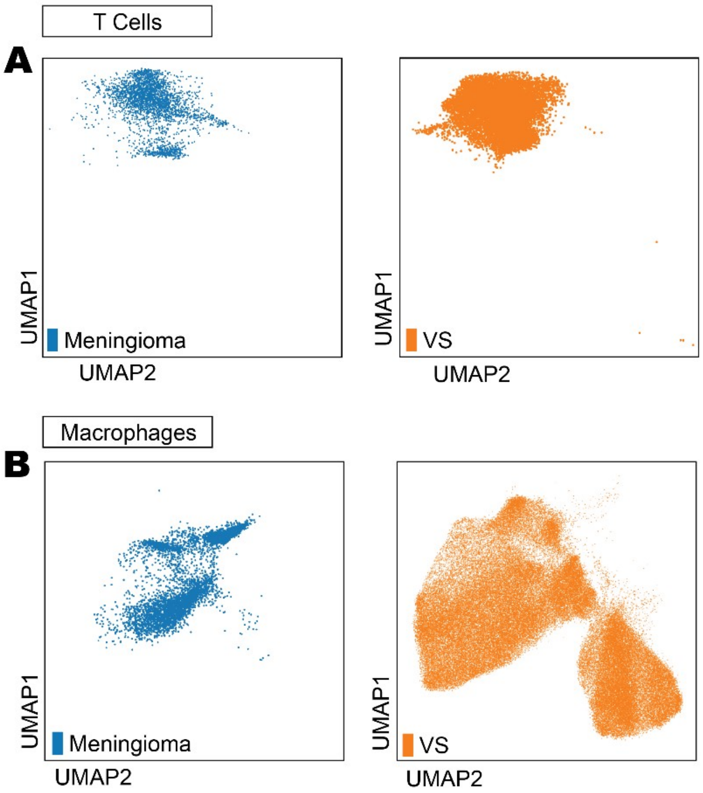

Supplementary Fig 9. Macrophages and T cells by tumour type. Single cell RNA sequencing data acquired from GSE183655 and GSE216783 from Gene Expression Omnibus containing meningioma (*n*=6) and VS samples (*n*=15), respectively. **(A)** Annotated T cell and **(B)** annotated macrophage populations visualised by UMAP.

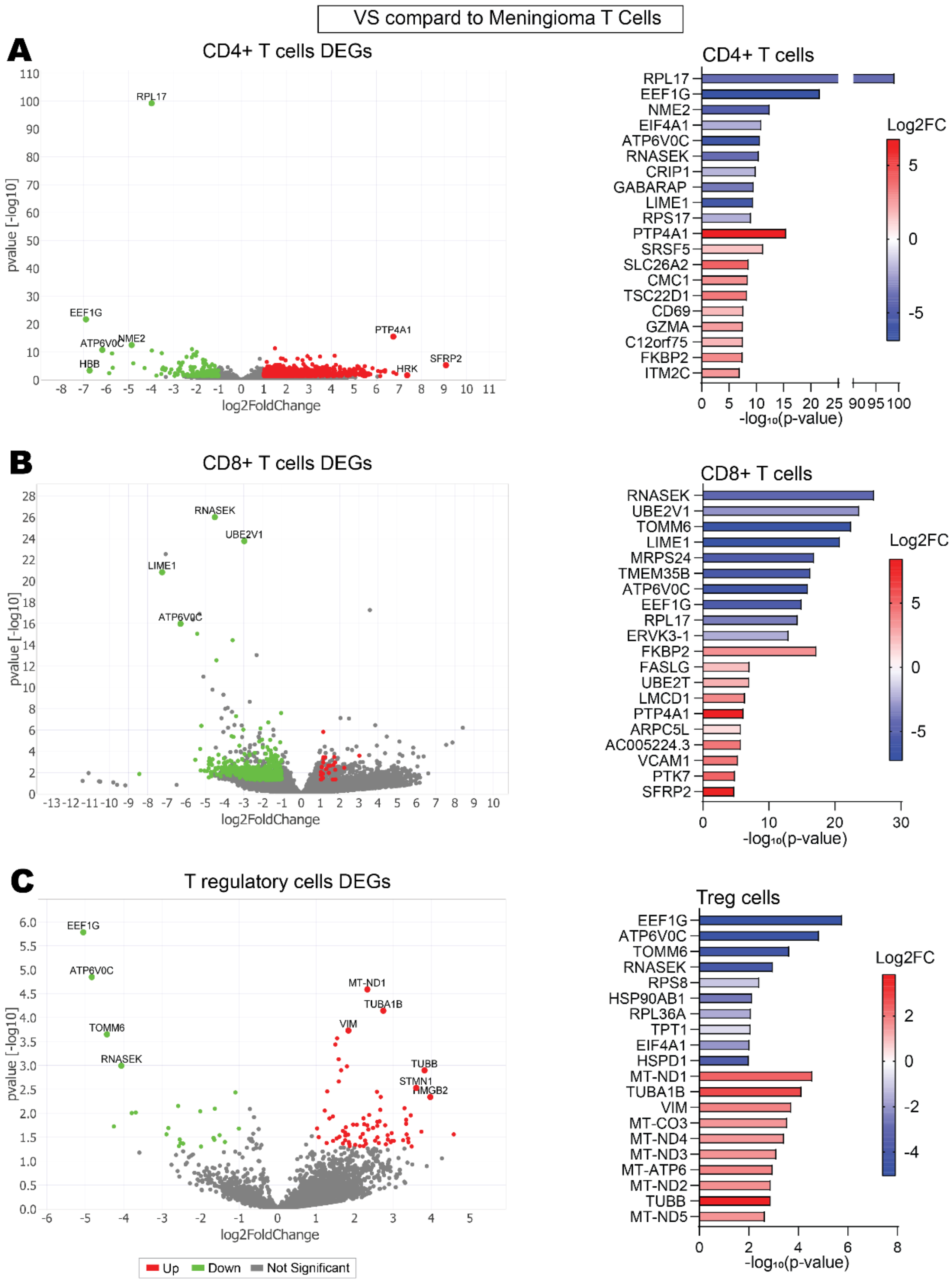

Supplementary Fig 10. VS and meningioma T cell subtypes display key differences in gene expression profiles. Pseudo-bulk from single cell RNA sequencing data from T cell subtypes **(A)** CD4+, **(B)** CD8+ and **(C)** T regulatory cells. Differentially expressed gene (DEG) volcano plot of pseudo-bulk VS compared to meningioma where DEGs were defined using the Wald test with Benjamini-Hochberg adjustment, significance as $p < 0.05$ and fold change >2 or <2. Top 10 significantly increased and decreased DEGs from pseudo-bulk VS CD4+, CD8+ and Treg cells compared to meningioma. Original data acquired from GSE183655 and GSE216783 from Gene Expression Omnibus containing meningioma (n=6) and VS samples (n=15), respectively.

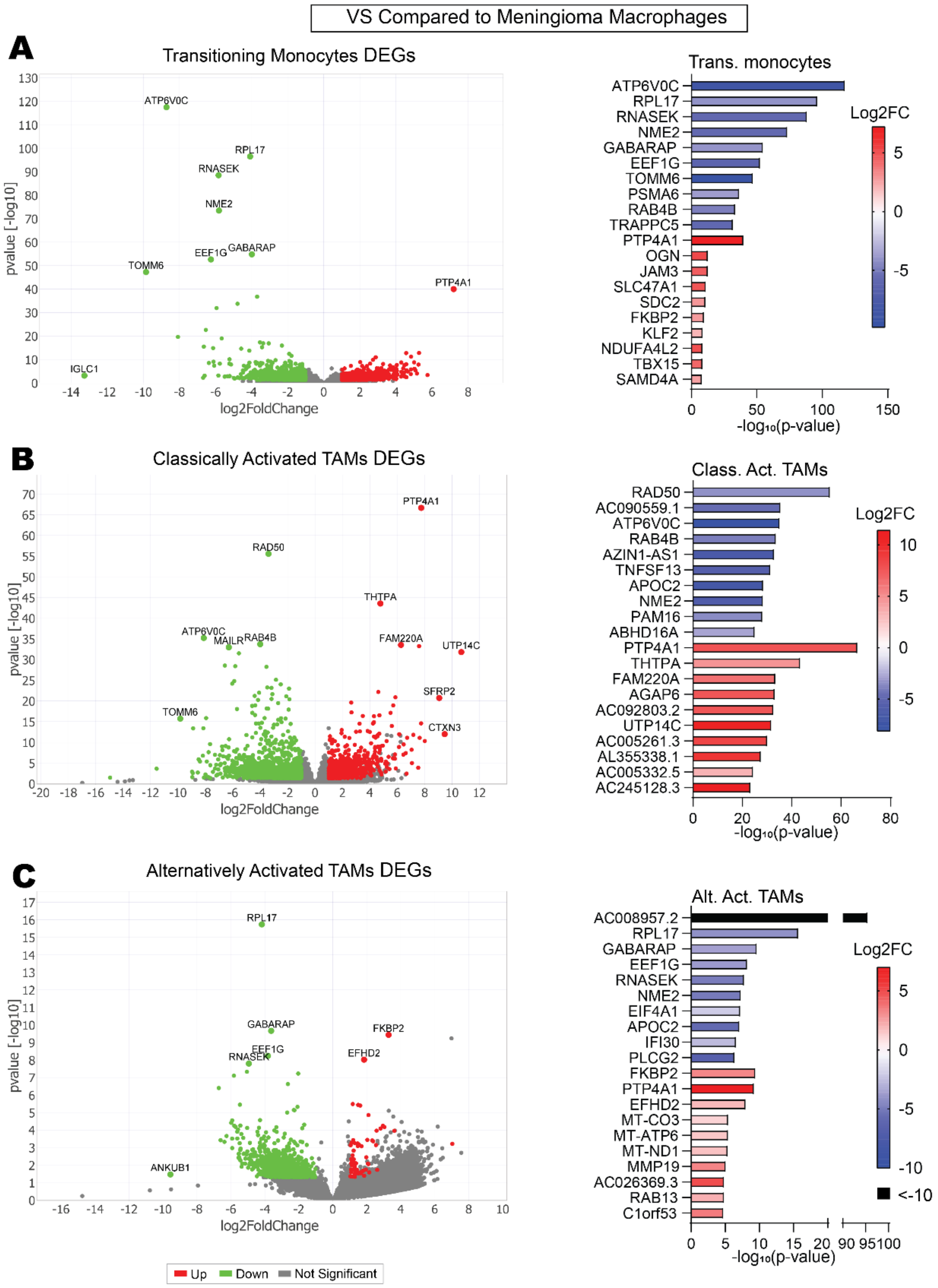

Supplementary Fig 11. VS and meningioma myeloid subtypes display key differences in gene expression profiles. Pseudo-bulk from single cell RNA sequencing data from myeloid subtypes **(A)** transitioning monocytes, **(B)** classically activated TAMs, and **(C)** alternatively activated TAMs. Differentially expressed gene (DEG) volcano plot of pseudo-bulk VS compared to meningioma where DEGs were defined using the Wald test with Benjamini-Hochberg adjustment, significance as $p < 0.05$ and fold change >2 or <2. Top 10 significantly increased and decreased DEGs from pseudo-bulk myeloid subtypes compared to meningioma. Original data acquired from GSE183655 and GSE216783 from Gene Expression Omnibus containing meningioma (*n*=6) and VS samples (*n*=15), respectively.
